# Efficacy and Safety of the Ketamine-Dexmedetomidine Combination in Adult Sedation and Anesthesia: A Systematic Review and Single-Arm Meta-Analysis of Randomized Controlled Trials

**DOI:** 10.7759/cureus.97432

**Published:** 2025-11-21

**Authors:** Alfredo B Junior, Paulo S Bendazzoli, Flavio W Ferreira Melo, Raissa M Porto Franco, Ezequiel M de Sousa Rocha, Raphael D Matos Lima, Enrico B Brondi

**Affiliations:** 1 Medical Affairs, Uninovafapi University Center, Teresina, BRA; 2 Anesthesiology and Perioperative Medicine, São Paulo State University (UNESP) Botucatu, Botucatu, BRA; 3 Anesthesiology, Federal University of Piauí, Teresina, BRA; 4 Anesthesiology, University Hospital of the Federal University of Uberlândia, Uberlândia, BRA; 5 Medical Affairs, Federal University of Maranhão, Pinheiros, BRA; 6 Medical Affairs, São Leopoldo Mandic College, São Paulo, BRA

**Keywords:** anesthesia, dexmedetomidine, ketamine, ketodex, randomized controlled trials

## Abstract

Background/objectives: Ketodex (KD) combines ketamine’s NMDA antagonism and sympathomimetic effects with dexmedetomidine’s selective α₂-agonism, offering synergistic sedation, analgesia, and hemodynamic stability while limiting drug-specific drawbacks. This study aimed to describe the efficacy and safety profile of KD for adult sedation and anesthesia through a single-arm meta-analysis approach.

Methods: PubMed, Cochrane Library, ScienceDirect, and Web of Science were searched for randomized controlled trials (RCTs) of KD in adults. Eligible studies reported safety or efficacy outcomes. Only the KD treatment arms were quantitatively pooled. Data on analgesia, recovery, satisfaction, and adverse events were pooled with random-effects models; heterogeneity was assessed by I².

Results: Twenty RCTs (874 participants) were included. Analgesia was consistent (mean visual analog score ≤2 for two to 12 hours). Recovery was rapid (mean 15 minutes) with a 40-minute post-anesthesia stay. Patient satisfaction reached 71-88%; physician satisfaction was 41% excellent and 54% adequate. Hemodynamics remained stable (mean arterial pressure 86.7 mmHg; heart rate 80 bpm; SpO₂ 98%). Adverse events varied: bradycardia occurred in 9% (95% CI 4-20%) but rose to 38% in breast cancer surgery. Hypotension affected 10% (95% CI 6-16%). Oxygen desaturation was usually 5-12% but reached 55% during painful emergency procedures (I² 61%). Post-operative nausea/vomiting affected 17% overall, exceeding 40% in abdominal or obstetric surgery. Neuropsychiatric effects hallucinations (7%), agitation (6%), nightmares (5%), and involuntary movements (15%) were more frequent in urgent cases.

Conclusions: In this single-arm synthesis, KD provides effective sedation and analgesia with stable vital signs and high satisfaction. Nonetheless, bradycardia, desaturation, and nausea remain clinically relevant and procedure-dependent, requiring careful patient selection and vigilant monitoring.

## Introduction and background

The combination of ketamine and dexmedetomidine, known as ketodex (KD), has emerged as a promising alternative for perioperative sedation and analgesia, particularly in opioid-free anesthesia, and has demonstrated efficacy and safety in adults undergoing a wide range of surgical and nonsurgical procedures [1-5].

This combination was developed to meet the need for anesthetic protocols that maximize analgesic and sedative effects while minimizing the adverse events inherent to each drug when used alone [6-9].

Initially introduced for short-duration procedures requiring stable sedation and rapid recovery, the KD combination has progressively expanded its role in adult anesthesia. Today, its use is well consolidated in ambulatory surgeries, minimally invasive interventions, and procedural sedation, where it offers reliable analgesia, hemodynamic stability, and reduced reliance on opioids or propofol. Its adoption has grown significantly in countries with a strong anesthesiology tradition, including the United States, Canada, and several European nations, as well as in emerging regions of Latin America and Asia, reflecting worldwide interest in its clinical applicability [10-12].

Ketamine provides intense dissociative analgesia, hemodynamic stability through sympathetic stimulation, and preservation of spontaneous respiratory drive. Dexmedetomidine, on the other hand, is a selective α2-adrenergic receptor agonist that decreases sympathetic outflow and leads to a unique sedative state known as "conscious sedation," anxiolysis, and analgesia, without causing significant respiratory depression. The combination of the KD creates a pharmacological synergy in which dexmedetomidine potentiates sedation and analgesia, whereas ketamine attenuates the cardiovascular depressant effects of dexmedetomidine [11,13-16].

The pharmacological synergy of ketamine and dexmedetomidine arises from their complementary mechanisms of action. Ketamine acts as a noncompetitive antagonist of N-methyl-D-aspartate (NMDA) receptors, thereby reducing glutamatergic excitatory transmission, central sensitization, and hyperalgesia. It also interacts with opioid (μ), monoaminergic, and muscarinic receptors, contributing to its analgesic and antidepressant properties. Dexmedetomidine exerts its sedative and analgesic effects mainly through presynaptic and postsynaptic α2-adrenergic receptor activation in the locus coeruleus and spinal cord dorsal horn, leading to inhibition of norepinephrine release, neuronal hyperpolarization, and suppression of sympathetic tone [4,5,11,13-16].

When combined, these agents act on distinct but complementary neural pathways: dexmedetomidine mitigates the sympathetic excitation and psychomimetic reactions of ketamine, while ketamine counterbalances the bradycardia and hypotension caused by dexmedetomidine. The result is a balanced therapeutic neuropharmacological interaction profile characterized by enhanced analgesia, improved hemodynamic stability, and minimal respiratory compromise, a multimodal synergy that strengthens both efficacy and safety in modern anesthetic practice, contributing to a more comfortable recovery [11,13-16].

Despite its growing use and promising results, the lack of universal protocols and the variability of indications highlight the need for robust studies to better define the optimal dosage, routes of administration, safety, and efficacy in different clinical settings [8,9,17,18].

Our findings suggest that the combination demonstrates a favorable balance between efficacy and safety across diverse clinical settings, although heterogeneity between studies highlights the need for further standardization. Therefore, this single-arm meta-analysis aims to comprehensively evaluate the efficacy and safety of a combination of ketamine and dexmedetomidine for sedation and anesthesia in adults, aiming to provide scientific evidence to guide clinical practice and increase the safety of anesthesia care [19-42].

## Review

Methods

This review was conducted in accordance with the essential reporting elements for systematic reviews and meta-analyses, as outlined in the Preferred Reporting Items for Systematic Reviews and Meta-Analyses (PRISMA) framework [43]. The protocol for this review was prospectively registered in the PROSPERO database under the registration number: CRD420251121026 [44].

Although only randomized controlled trials (RCTs) were included, this review was designed and analyzed as a single-arm meta-analysis. This methodological decision was based on the substantial heterogeneity among comparator groups across the included RCTs, which used different anesthetic regimens, doses, and perioperative contexts. Consequently, direct between-group comparisons would not yield methodologically valid or clinically interpretable results. Therefore, the analysis focused exclusively on outcomes from KD-treated arms, aiming to describe its efficacy and safety profile across diverse adult populations and procedural types.

Search Strategy

A comprehensive and systematic search was conducted in the PubMed, Cochrane Library, ScienceDirect, and Web of Science databases to identify relevant publications up to August 20, 2025. The search strategy was designed to identify RCTs evaluating the efficacy, safety, and perioperative outcomes associated with the use of the KD in adult patients undergoing sedation or anesthesia for diagnostic or therapeutic procedures. Medical subject heading (MeSH) terms and free-text keywords such as ‘ketamine,’ ‘dexmedetomidine,’ ‘ketodex,’ ‘sedation,’ ‘anesthesia,’ and ‘randomized controlled trial’ were used, combined with Boolean operators (AND, OR, NOT) to ensure the identification of studies relevant to the outcomes of interest. To enhance replicability, the complete search strings used in each database (including filters and field codes) can be seen in Table 1.

**Table 1:** Search terms and strategy used for database search This table shows the complete search strategy applied to each electronic database. MeSH terms and free-text keywords were combined using Boolean operators (AND, OR, NOT) to identify randomized controlled trials assessing the efficacy, safety, and perioperative outcomes of the ketamine–dexmedetomidine combination in adult patients undergoing sedation or anesthesia for diagnostic or therapeutic procedures.

Database	Search terms / Strategy
PubMed	(“Ketamine”[Mesh] OR “ketamine”[tiab]) AND (“Dexmedetomidine”[Mesh] OR “dexmedetomidine”[tiab] OR “ketodex”[tiab]) AND (“Sedation”[Mesh] OR “Anesthesia”[Mesh] OR “sedation”[tiab] OR “anesthesia”[tiab]) AND (“Adult”[Mesh] OR “adult”[tiab]) AND (“Randomized Controlled Trial”[Publication Type] OR “RCT”[tiab] OR “randomized”[tiab]) NOT (animals[mh] NOT humans[mh])
Cochrane Library	(“ketamine” OR “dexmedetomidine” OR “ketodex”) AND (“sedation” OR “anesthesia”) AND (“adult”) AND (“randomized controlled trial” OR “RCT”)
ScienceDirect	(“ketamine” OR “dexmedetomidine” OR “ketodex”) AND (“sedation” OR “anesthesia”) AND (“adult”) AND (“randomized controlled trial”))
Web of Science	((“ketamine” OR “dexmedetomidine” OR “ketodex”) AND (“sedation” OR “anesthesia”) AND (“adult”) AND (“randomized controlled trial”))

To ensure consistency, studies were not excluded based on differences in comparator interventions, as only data from KD intervention arms were extracted for quantitative synthesis.

Inclusion and Exclusion

Only RCTs enrolling adult patients undergoing sedation or anesthesia for diagnostic or therapeutic procedures, regardless of the nature of the procedure, were included. No restrictions were applied regarding sex. To ensure data robustness, only studies with at least five participants per group were considered. Studies were included if ketamine or esketamine combined with dexmedetomidine (ketodex) was administered for sedation or anesthesia. There were no restrictions on dosage or route of administration.

No comparisons with other anesthetic or sedative regimens were required, as the review focused exclusively on the evaluation of KD. This approach was chosen to minimize confounding from heterogeneous comparator arms and to maintain methodological consistency in estimating pooled outcomes. The eligibility criteria for study selection were as follows: (1) randomized controlled trial design, even if single-arm, (2) availability of the full text, (3) publication in peer-reviewed journals, and (4) no restrictions regarding language or publication date.

Studies were excluded if they were (1) observational studies, case reports, case series, or non-randomized trials; (2) narrative or systematic reviews, meta-analyses, editorials, letters to the editor, or conference abstracts without full-text availability; (3) studies conducted in animal models or in vitro. Duplicate publications, studies with overlapping data, or those lacking clear reporting of outcomes of interest or sufficient information on participant numbers were also excluded. When multiple articles originated from the same clinical trial, all were assessed collectively. The earliest publication was considered the primary reference; however, data extraction was based on the most complete and updated report available for each outcome.

Outcomes Definitions 

The outcomes assessed in this review included adverse events and clinical parameters related to the use of KD in adult patients undergoing sedation or anesthesia across various clinical settings and procedure types. Cardiovascular outcomes, respiratory outcomes, neuropsychiatric outcomes, and gastrointestinal events were analyzed. Clinically relevant outcomes included recovery time, length of stay in the post-anesthesia care unit (PACU), and postoperative pain intensity, evaluated at three time points (two hours, six hours, and 12 hours after KD administration). Additional outcomes included physician and patient satisfaction. When available, mean values and standard deviations of physiological parameters, including mean arterial pressure (MAP), heart rate (HR), respiratory rate (RR), and peripheral oxygen saturation (SpO₂), were extracted to assess hemodynamic and ventilatory stability.

Study Selection and Data Extraction

Data extraction was performed manually by two independent reviewers (A.B. and R.L.), who conducted both the literature search and study selection in a blinded manner on the basis of the predefined eligibility criteria. All identified records underwent an initial screening of titles and abstracts, followed by full-text assessment of potentially eligible studies. Throughout the process, all data were cross-checked among the reviewers, in cases of disagreement, decisions were resolved by consensus, with the involvement of a third reviewer (P.S.) when necessary.

The extracted information included the following: publication characteristics (first author, year, country of origin, study design); participant demographics (group size, mean age, weight, sex, body mass index (BMI), and ASA classification); procedure details (type, mean duration, and anesthetic regimen used); administered doses of ketamine and dexmedetomidine; and predefined clinical outcomes, categorized accordingly. Units of measurement were standardized across studies: dexmedetomidine doses were reported in micrograms (μg or μg/kg/h) and ketamine/esketamine in milligrams (mg or mg/kg).

All the data were extracted exclusively from information available in the original publications, including the main text, tables, figures, and supplementary materials. It was not necessary to contact the study authors. A summary of the extracted data is presented in the corresponding tables.

Assessment of Risk of Bias (ROB)

The assessment of methodological bias in the included studies was performed using the Cochrane Collaboration Risk of Bias (ROB) 2.0 tool [45], which is specifically designed for randomized clinical trials. This assessment encompassed six domains: (1) the randomization process, (2) deviations from intended interventions, (3) missing outcome data, (4) outcome measurement, (5) selection of reported results, and (6) the overall risk of bias. Each domain was categorized as low risk, high risk, or presenting some concerns.

Two authors (A.B. and R.L.) independently conducted the assessment, and any disagreements were resolved by a third reviewer (P.S.). The RoB 2 tool was applied to evaluate potential biases related to study design, methodological conduct, and outcome reporting.

Data Analyses

Continuous data from the evaluated population, including mean values, standard deviations, and proportions, were extracted. On the basis of this information, 95% confidence intervals (CIs) were estimated for the outcomes of interest. As the analysis involved a single group only, no between-group comparisons or mean difference calculations were performed [46].

Pooled estimates were obtained via a random-effects model, generating proportion estimates adjusted for heterogeneity. The random-effects model was selected a priori to account for expected clinical and methodological variability across included trials, given the heterogeneity of surgical procedures and patient populations.. Heterogeneity was assessed via Cochran’s Q test and the I² statistic, which quantifies the degree of inconsistency across results. The corresponding p value was also estimated to determine statistical significance. The interpretation of I² values followed conventional thresholds: 0-40% indicates negligible heterogeneity, 30-60% indicates moderate heterogeneity, 50-90% indicates substantial heterogeneity, and 75-100% indicates considerable heterogeneity.

When I² values exceeded 75%, pooled estimates were interpreted with caution, and results were further explored through sensitivity analyses excluding outlier studies. Whenever possible, subgroup analyses were performed to evaluate procedure-related differences (minor vs. major interventions), and potential sources of heterogeneity were investigated via exploratory meta-regression.

P-values were reported consistently alongside 95% CIs to facilitate the interpretation of statistical and clinical significance. All the statistical analyses were performed via R software, version 4.3.2 (R Foundation for Statistical Computing, Vienna, Austria). The ‘meta’ package was used to conduct the meta-analysis, and the results are presented via forest plots. All R scripts used for analysis are available upon request to allow complete reproducibility of the analytic workflow.

Results

Study Selection

**Figure 1:**
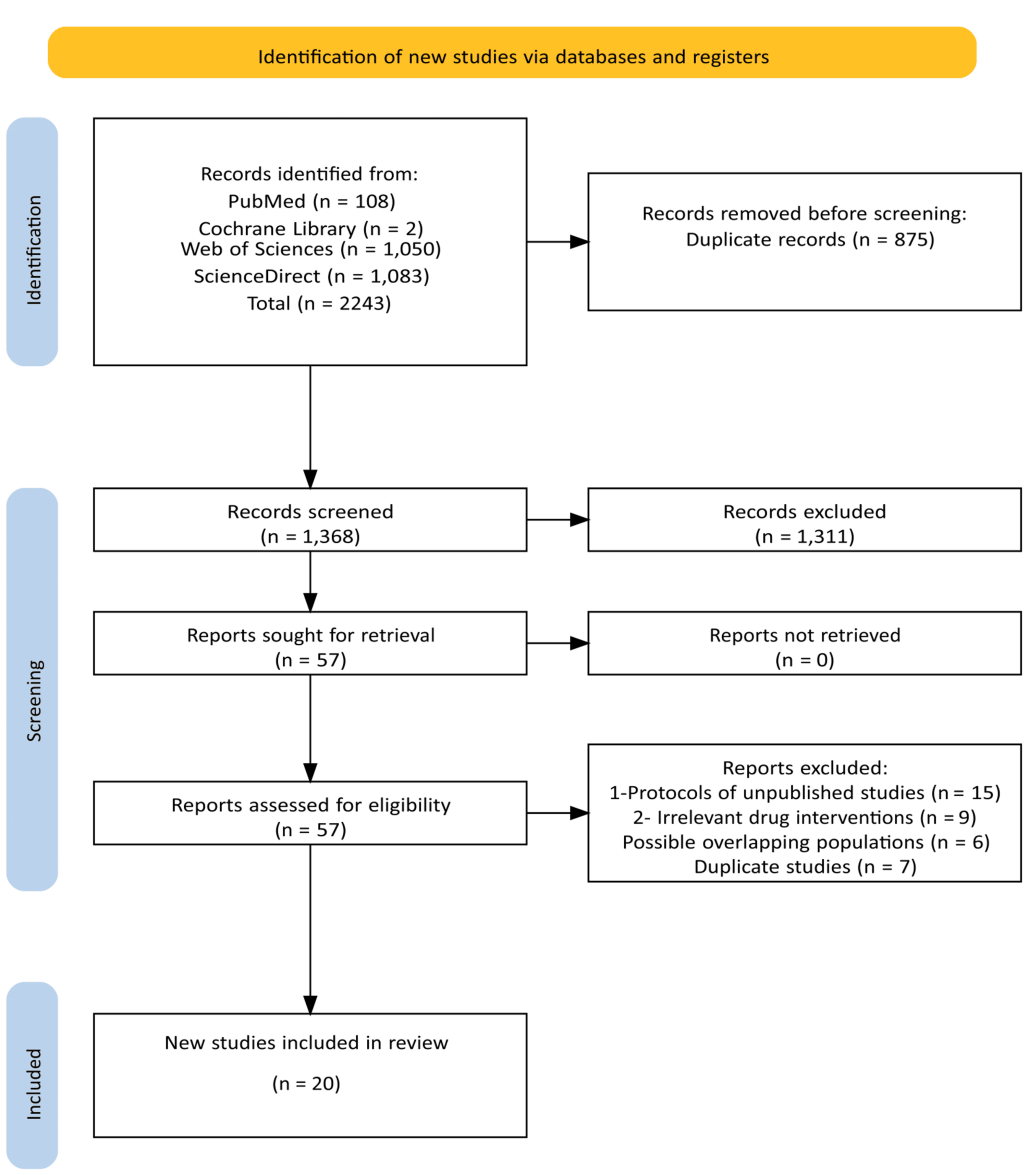
PRISMA flow diagram of study selection PRISMA: Preferred Reporting Items for Systematic reviews and Meta-Analyses

The initial search yielded 2,243 records across PubMed (n = 108), Cochrane Library (n = 2), Web of Science (n = 1,050), and ScienceDirect (n = 1,083). After removing 875 duplicates and excluding 1,311 studies based on title and abstract screening, 57 articles were selected for full-text review, as detailed in Figure 1. Of these, 20 randomized controlled trials fulfilled the pre-specified eligibility criteria and were included in the final quantitative analysis. The main reasons for exclusion were unpublished study protocols (n = 15), irrelevant pharmacological interventions (n = 9), potential sample overlap (n = 6), and data duplication (n = 7). The reasons for the exclusion of the remaining articles are reported in Figure 1.

Study Characteristics

A total of 874 patients were included in the 20 randomized controlled trials, of whom 426 were men and 448 women. The mean age was 46.4 ± 10.7 years, with a mean BMI of 24.7 ± 2.9 kg/m². When reported, the average weight was 67.5 ± 12.1 kg. The anesthetic management described across the studies commonly demonstrated that dexmedetomidine was mostly administered as an initial infusion ranging from 0.5 to 1 µg/kg over approximately 10 minutes. Ketamine or esketamine was given either as a single or continuous infusion, with initial doses ranging from 0.3 to 1 mg/kg, adjusted according to each study protocol. Median bolus doses were 0.9 μg/kg (IQR: 0.5-1.0) for dexmedetomidine and 0.5 mg/kg (IQR: 0.375-0.625) for ketamine/esketamine. Mean procedure duration was 51.2 ± 32.4 minutes, varying from less than 10 minutes in minor procedures to over 160 minutes in major surgeries. Baseline characteristics and anesthetic management are shown in detail in Table 2.

**Table 2:** Baseline characteristics of included studies Study ID: identifier, N: sample size, M: mean, SD: standard deviation, BMI: body mass index, kg/m²: kilograms per square meter, Min: minutes, ERCP: endoscopic retrograde cholangiopancreatography, CABG: coronary artery bypass grafting

Study ID	Country	Sample Size (N)	Mean age (year) M (SD)	Male/Female	BMI (Kg/m2) M (SD)	Procedure	Anesthesic Strategy	Procedure duration (min) M (SD)
Algharabawy et al. (2021) [25]	Egypt	35	44.31 ± 6.76	25 / 10	27.9 ± 1.9	Upper gastrointestinal endoscopy	Initial infusion: 1 mg/kg ketamine and 1 μg/kg dexmedetomidine over 10 min; Maintenance infusion: 0.1 mg/kg/h ketamine and 0.1 μg/kg/h dexmedetomidine	15.26 ± 3.61
Azizkhani et al. (2021) [29]	Iran	31	39 ± 18	24 / 7	-	Painful procedures in the emergency department	Single IV bolus: ketamine 1 mg/kg + dexmedetomidine 0.7 μg/kg diluted in 0.9% saline (20 mL syringe) over 4 min	12 ± 3
Chun et al. (2016) [22]	South Korea	25	50.6 ± 9.8	3 / 22	23.2 ± 4.0	Chemoport Insertion	Initial infusion: dexmedetomidine 1 μg/kg over 10 min + ketamine 0.5 mg/kg; Maintenance: dexmedetomidine 0.2–1.0 μg/kg/h, rescue ketamine 0.5 mg/kg	6.98 ± 6.33
Canpolat et al. (2012) [1]	Turkey	30	29.33 ± 18.01	21 / 9	-	Burn Dressing Changes	Initial infusion: ketamine 0.5 mg/kg + dexmedetomidine 1 μg/kg over 10 min; Maintenance: ketamine 0.5 mg/kg + dexmedetomidine 1 μg/kg continuous	11.03 ± 4.79
El Sharkawy et al. (2019) [32]	Egypt	30	43.6 ± 11.6	17 / 13	27.9 ± 2.5	Awake Fiber‐optic Intubation	Initial infusion: ketamine 0.5 mg/kg + dexmedetomidine 1 μg/kg over 10 min; Maintenance: ketamine 0.5 mg/kg + dexmedetomidine 1 μg/kg	0.98 ± 0.10
Goyal et al. (2016) [31]	India	41	60.4 ± 13.8	30 / 11	24 ± 3.2	Elective Retrograde Cholangiopancreatography	Initial: dexmedetomidine 0.5 mg/kg + ketamine 1 mg/kg (2 divided boluses, 30 s); Maintenance: dexmedetomidine 0.5 mg/kg/h + ketamine 1–2 mg/kg/h	41.8 ± 15.6
Hu et al. (2024) [20]	China	55	45.17 ± 10.26	25 / 30	-	Laparoscopic Cholecystectomy	Initial: dexmedetomidine 1 μg/kg over 10 min + esketamine 0.4 mg/kg; Maintenance: dexmedetomidine 0.5 μg/kg/h + esketamine 0.1 mg/kg/h	-
Huang et al. (2023)a [19]	China	45	51.8 ± 7.3	- / -	23.1 ± 2.1	Radical Mastectomy	Initial: dexmedetomidine 0.5 μg/kg + esketamine 0.5 mg/kg; Maintenance: dexmedetomidine 0.4 μg/kg/h + esketamine 2 μg/kg/min	74.3 ± 5.6
Huang et al. (2023)b [19]	China	45	50.4 ± 6.8	- / -	24.0 ± 2.1	Radical Mastectomy	Initial: dexmedetomidine 0.5 μg/kg + esketamine 0.5 mg/kg; Maintenance: dexmedetomidine 0.4 μg/kg/h + esketamine 4 μg/kg/min	74.8 ± 5.2
Lin et al. (2023) [26]	China	22	64.59 ± 9.62	14 / 8	22.66 ± 3.12	Lung tumor PRFA	Initial: dexmedetomidine 1 μg/kg over 10 min + esketamine 0.2 mg/kg; Maintenance: esketamine 0.1 mg/kg/h + dexmedetomidine 0.6 μg/kg/h	74.91 ± 24.07
Makwana et al. (2022) [21]	India	33	37.11 ± 12.64	- / -	22.23 ± 2.31	Upper limb surgeries	Initial: ketamine 0.5 mg/kg + dexmedetomidine 0.5 μg/kg over 10 min; Maintenance: ketamine 0.3 mg/kg/h + dexmedetomidine 0.3 μg/kg/h	-
Massoth et al. (2021) [27]	Germany	76	30.0 ± 12.34	- / -	24.2 ± 2.7	Gynaecological laparoscopy	Initial: dexmedetomidine 0.6 μg/kg over 7 min + esketamine 0.15 mg/kg; Maintenance: dexmedetomidine 0.3 μg/kg/h + esketamine 0.15 mg/kg/h	80.5 ± 16.4
Modir et al. (2021) [23]	Iran	30	40.23 ± 9.57	16 / 14	-	Cystoscopy	Single bolus: ketamine 0.5 mg/kg + dexmedetomidine 1 μg/kg	13.23 ± 1.85
Mogahd et al. (2017) [2]	Egypt	35	53.5 ± 4.9	18 / 17	27.3 ± 2.6	CABG Surgery	Initial: ketamine 1 mg/kg + dexmedetomidine 1 μg/kg over 20 min; Maintenance: ketamine 0.25 mg/kg/h + dexmedetomidine 0.2–0.7 μg/kg/h	-
Saini et al. (2020) [30]	India	50	43.7 ± 9.87	19 / 31	-	Laparoscopic Cholecystectomy	Initial: ketamine 0.5 mg/kg + dexmedetomidine 0.4 μg/kg/h; Maintenance: rescue ketamine 0.3 mg/kg	54.8 ± 6.67
Singh et al. (2022) [3]	India	42	43.5 ± 15.4	22 / 20	20.7 ± 1.9	ERCP	Initial: dexmedetomidine 1 μg/kg over 10 min + ketamine 0.5 mg/kg; Maintenance: ketamine 0.5 mg/kg/h + dexmedetomidine 0.5 μg/kg/h; Rescue: ketamine 10 mg	46.7 ± 18.5
Singh et al. (2023) [24]	India	30	50.63 ± 12.61	6 / 24	-	ERCP	Initial: dexmedetomidine 1 μg/kg + ketamine 1 mg/kg; Maintenance: dexmedetomidine 0.5 μg/kg/h + ketamine 0.5 mg/kg as rescue	-
Wang et al. (2025) [5]	China	80	54.7 ± 9.6	49 / 21	22.6 ± 2.3	Laparoscopic Major Abdominal Surgery	Initial: esketamine 0.2–0.5 mg/kg/h + dexmedetomidine 0.2–0.7 μg/kg/h over 10 min post-induction	163.7 ± 61.5
Xue et al. (2024) [12]	China	30	57.9 ± 6.3	18 / 12	25.5 ± 3.0	Shoulder arthroscopy	Initial: dexmedetomidine 0.8–1 μg/kg over 10 min + esketamine 0.3 mg/kg; Maintenance: dexmedetomidine 0.3–0.5 μg/kg/h + esketamine 0.15 mg/kg/h	106.8 ± 17.2
Yang et al. (2024) [28]	China	76	33.3 ± 5.2	0 / 76	26.6 ± 2.8	Cesarean (after cord clamping)	Single bolus: esketamine 0.3 mg/kg + dexmedetomidine 0.5 μg/kg	47.7 ± 18.5
Yeter et al. (2021) [18]	Turkey	30	40 ± 17	15 / 15	25.9 ± 5.7	Electroconvulsive therapy	Initial: dexmedetomidine 1 μg/kg over 10 min + ketamine 1 mg/kg; Maintenance: ketamine 0.5 mg/kg as rescue	-

Clinical Effectiveness

**Figure 2:**
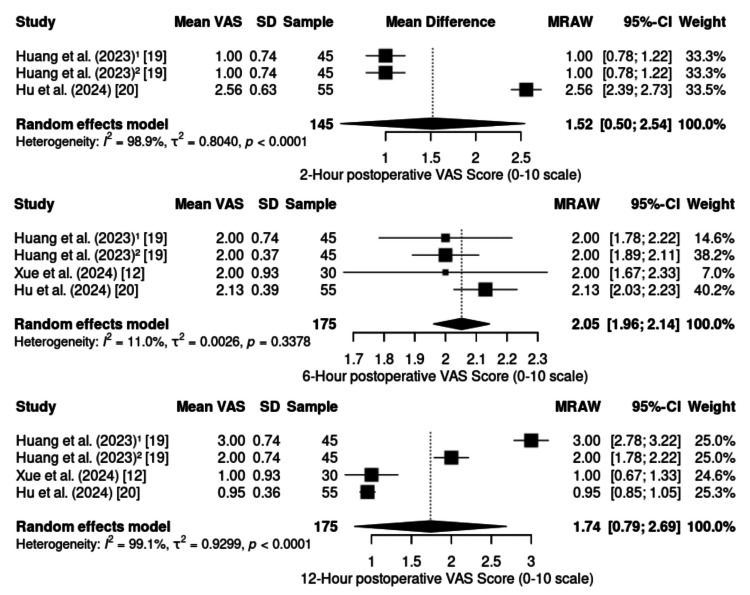
Forest plot of postoperative visual analog scale (VAS) score. CI: confidence interval; SD: standard deviation; MRAW: mean difference – raw

Postoperative pain and analgesic effectiveness:* *Postoperative pain was assessed at different time points via the visual analog scale (VAS), in addition to its overall incidence during the postoperative period (Figure 2). Three studies (n = 145) reported a pooled mean VAS score of 1.52 (95% CI: 0.50--2.54), with high heterogeneity (I² = 98.9%; p < 0.0001), ranging from 1.0 in radical mastectomy [19] to 2.56 in laparoscopic cholecystectomy [20]. At six hours, four studies (n = 175) reported a pooled mean of 2.05 (95% CI: 1.96--2.14), with low heterogeneity (I² = 11.0%; p = 0.3378) and minimal variation between 2.0 (radical mastectomy) [19] and 2.13 (laparoscopic cholecystectomy) [20]. At 12 hours, four studies (n = 175) showed a pooled mean of 1.74 (95% CI: 0.79--2.69), again with substantial heterogeneity (I² = 99.1%; p < 0.0001), ranging from 0.95 in laparoscopic cholecystectomy [20] to 3.0 in radical mastectomy [19].

**Figure 3:**
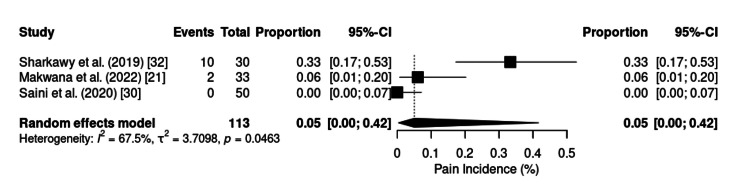
Forest plot of incidence of postoperative pain. CI: confidence interval

The overall incidence of postoperative pain (Figure 3), assessed as a dichotomous outcome in three studies (n = 113), was estimated at 5% (95% CI: 0%-42%; I² = 67.5%; p = 0.0463) via a random effects model. Lower pain scores and incidences are generally observed in minimally invasive procedures such as shoulder arthroscopy [21] and laparoscopic cholecystectomy [20], whereas higher values are recorded in major surgeries such as radical mastectomy [19], particularly when associated with higher continuous infusion doses of ketamine (≥0.5 mg/kg) and dexmedetomidine (≥0.5 μg/kg).

Physician and Patient Satisfaction

**Figure 4:**
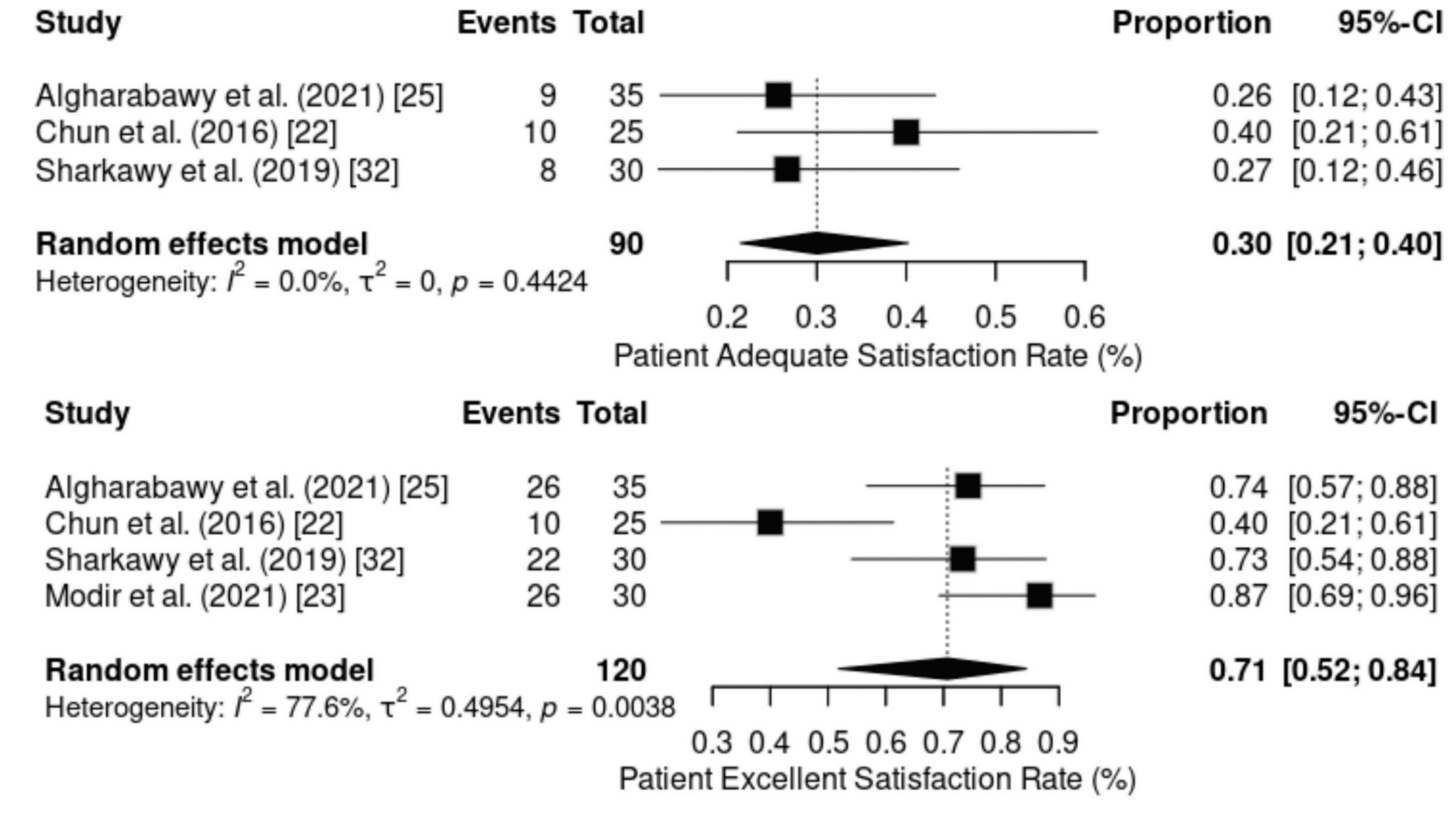
Forest plot of incidence of patient satisfaction. CI: confidence interval

**Figure 5:**
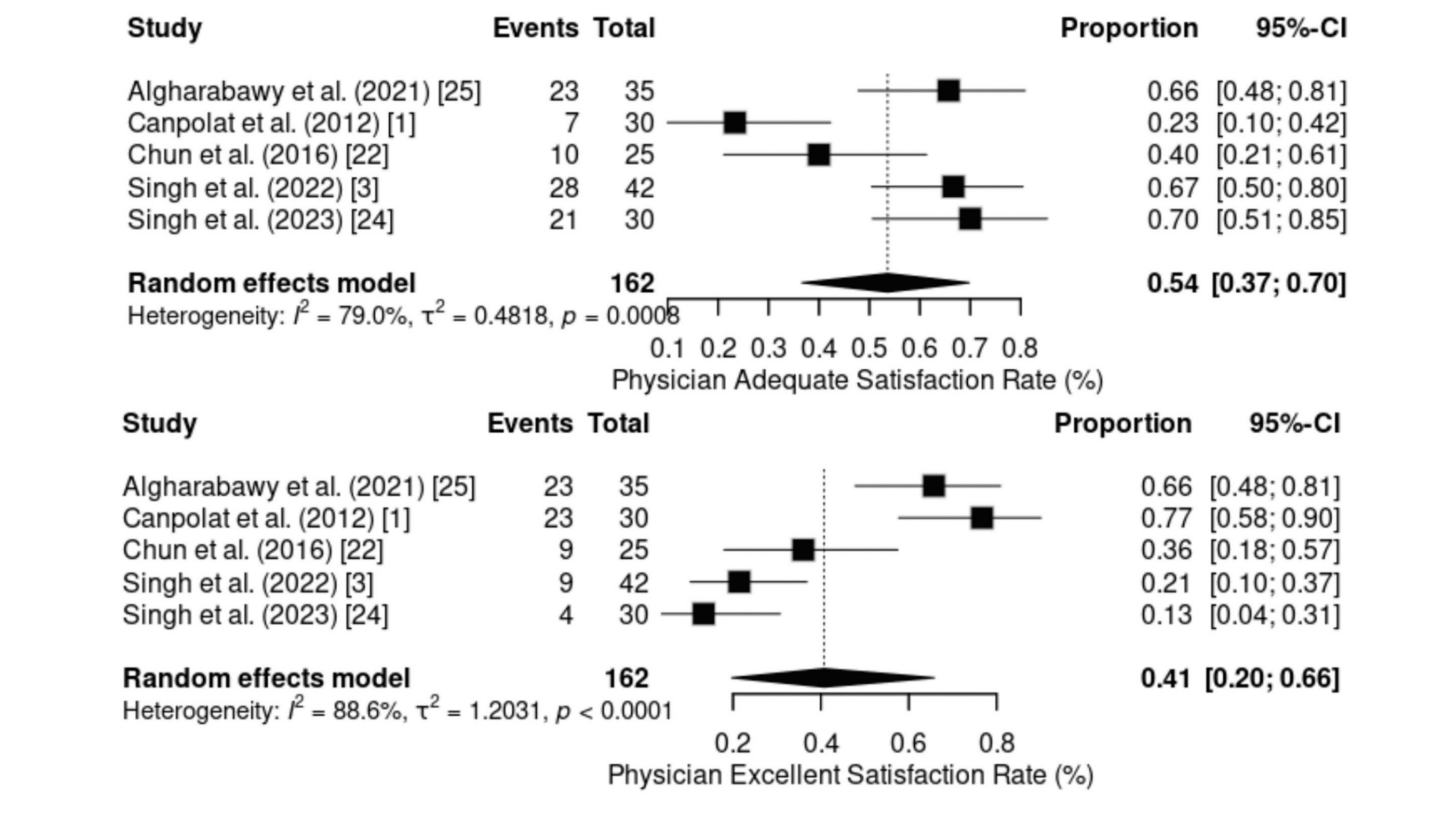
Forest plot of incidence of physician satisfaction. CI: confidence interval

Satisfaction with the use of the KD combination was analyzed on the basis of “excellent” and “adequate” ratings from both the patients’ and physicians’ perspectives (Figure 4 and Figure 5).

Excellent patient satisfaction was evaluated in four RCTs (n = 120). Substantial heterogeneity was observed (p = 0.0038; I² = 77.6%). The pooled incidence was 71% (95% CI: 52%-84%), with rates ranging from 40% during chemoport insertion [22] to 87% during cystoscopy procedures [23].

Adequate patient satisfaction was reported in three studies (n = 90), with a pooled proportion of 30% (95% CI: 21%-40%) and no evidence of heterogeneity (p > 0.05; I² = 0%), indicating consistency across findings. These results suggest that most patients rated their anesthetic experience positively, with a predominance of “excellent” ratings over “adequate.”

From the physicians’ perspective, five studies (n = 162) evaluated excellent satisfaction, with a pooled incidence of 41% (95% CI: 20%-66%). Very high heterogeneity was identified (I² = 88.6%; p < 0.0001), with considerable variation across procedures from 13% in endoscopic retrograde cholangiopancreatography (ERCP) [24] to 77% in burn dressing applications [1].

Adequate satisfaction among physicians, assessed in the same studies (n = 162), represented a pooled proportion of 54% (95% CI: 37%-70%), with high heterogeneity (p = 0.0008; I² = 79%). The values ranged from 23% [1] to 70% [24], suggesting that, unlike patients, physicians tend to favor “adequate” classification over “excellent”, possibly reflecting a more conservative stance or specific clinical expectations regarding the effectiveness of the protocol.

Recovery and Post-anesthesia Care Stay Times

**Figure 6:**
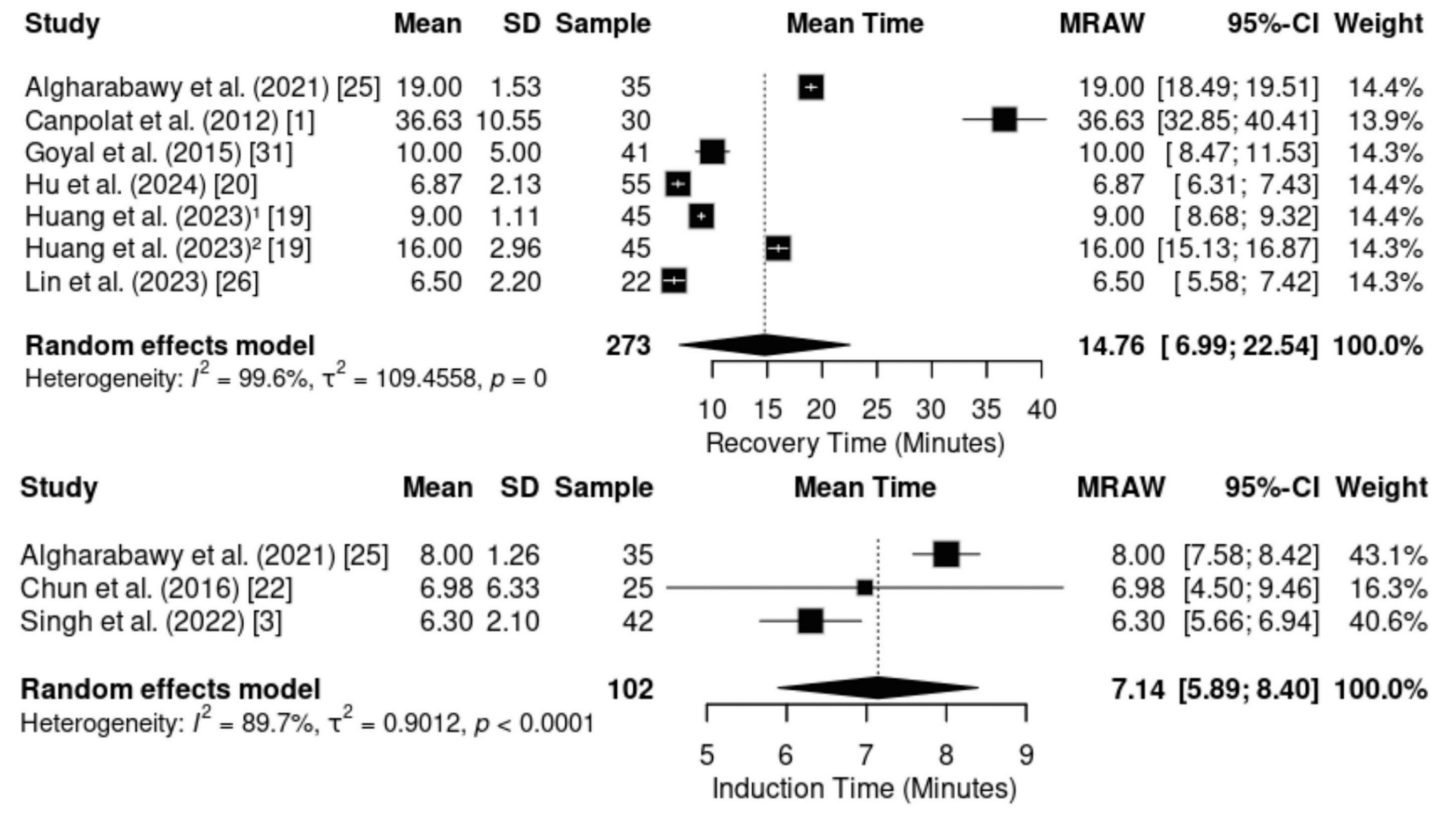
Forest plot of induction time and recovery time. CI: confidence interval; SD: standard deviation; MRAW: mean difference – raw

Induction time was assessed in three studies (n = 102), revealing a pooled mean of 7.14 minutes (95% CI: 5.89-8.40), despite substantial heterogeneity (I² = 89.7%, p < 0.0001) (Figure 6). Individual means ranged from 6.30 minutes [24] to 8.00 minutes [25]. Despite differences in the type of procedure, the induction times consistently remained below 10 minutes. This pattern reinforces the rapid onset profile of KD sedation, even across diverse clinical scenarios.

Recovery time was reported in seven studies (n = 273), with a pooled mean of 14.76 minutes (95% CI: 6.9922.54; I² = 98.5%) (Figure 6). Although heterogeneity was high, the overall trend indicated efficient recovery, ranging from just 6.5 minutes [26] to 36.6 minutes [1]. This contrast suggests that procedural complexity and the intensity of nociceptive stimuli exert a greater influence than the pharmacodynamics of the KD combination itself.

**Figure 7:**
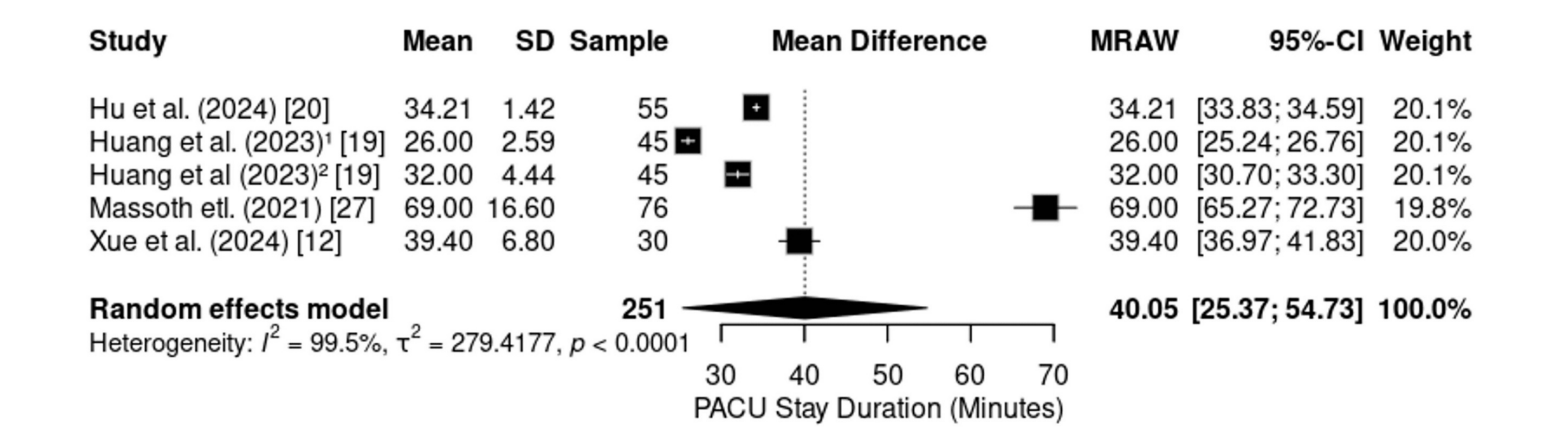
Forest plot of length of stay in the PACU. PACU: post-anesthesia care unit; CI: confidence interval; SD: standard deviation; MRAW: mean difference – raw

The length of stay in the PACU was reported in five studies (n = 251), with a pooled mean of 40.05 minutes (95% CI: 25.37-54.73; I² = 99.5%) (Figure 7). Substantial variability was observed, from 26 minutes [19] to 69 minutes [27]. Thus, although KD provides a predictably stable recovery, the total PACU stay appears to be strongly influenced by the nature and invasiveness of the surgical procedure rather than by the anesthetic regimen itself.

Safety Profile and Adverse Events

**Figure 8:**
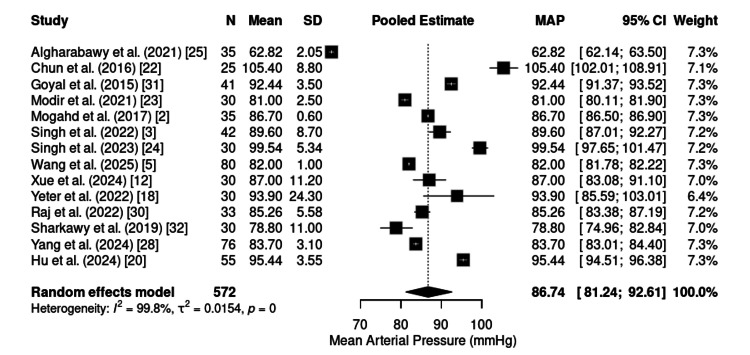
Forest plot of intraoperative MAP. MAP: mean arterial pressure; CI: confidence interval; SD: standard deviation

Hemodynamic stability:* *The MAP was reported in 15 studies, encompassing a total of 632 patients. The pooled mean was 86.74 mmHg (95% CI: 81.24-92.61), with significant heterogeneity across studies (I² = 99.8%) (Figure 8). Despite variations in procedures and participant characteristics, the values remained within the physiological range, reflecting adequate maintenance of hemodynamic stability.

**Figure 9:**
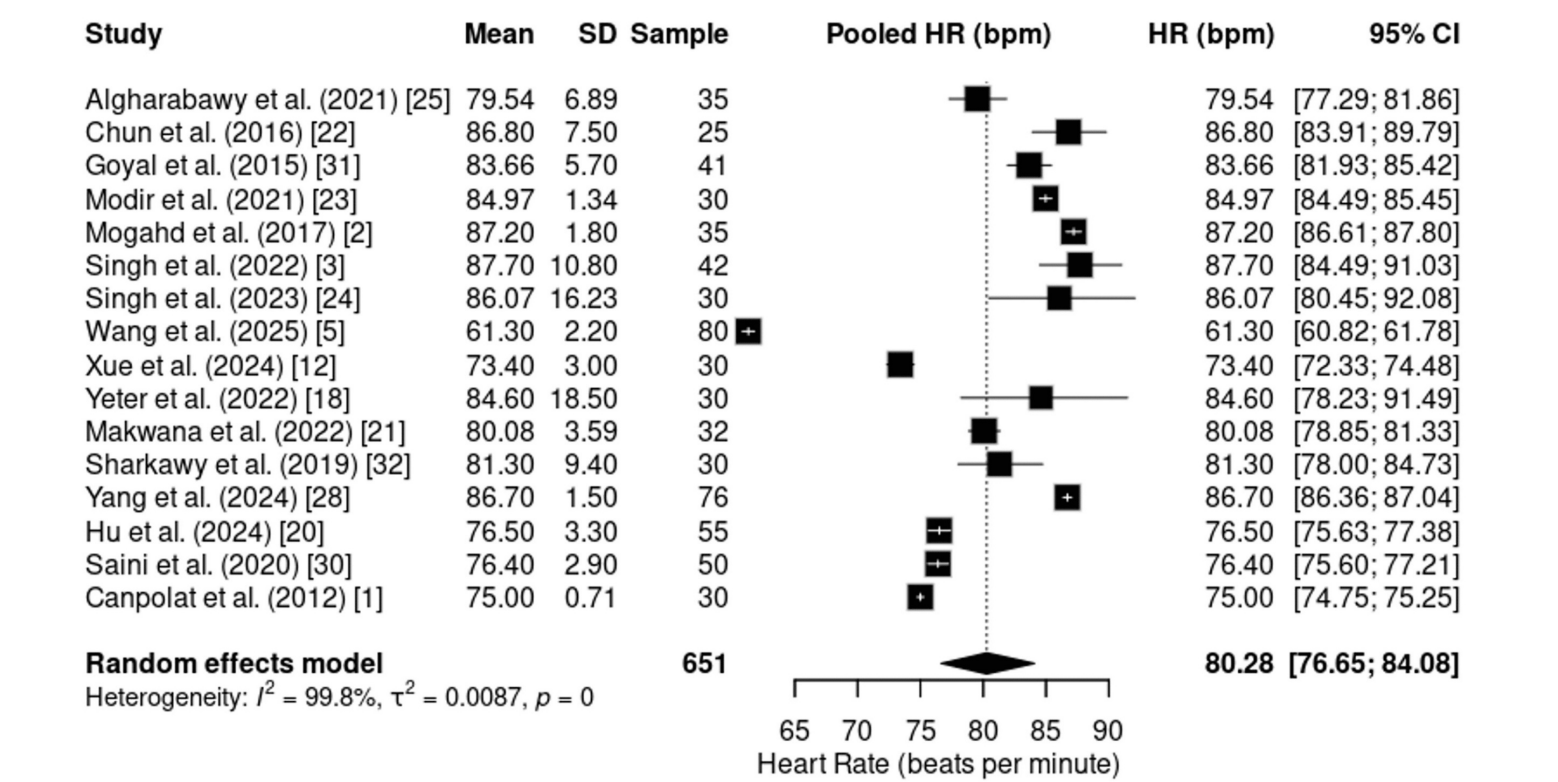
Forest plot of intraoperative HR. HR: heart rate; CI: confidence interval; SD: standard deviation

HR was reported in 16 studies, totaling 657 patients. The combined mean was 80.28 bpm (95% CI: 76.65-84.08) (Figure 9), with high heterogeneity (I² = 99.8%). Nevertheless, the values observed indicate chronotropic stability during procedures, with no predominance of clinically relevant bradycardia or tachycardia.

**Figure 10:**
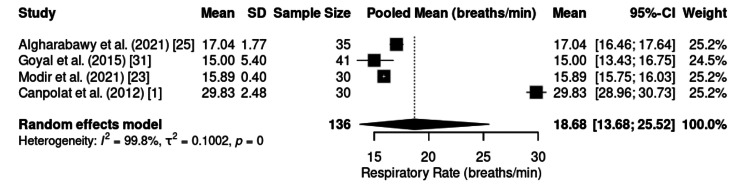
Forest plot of intraoperative RR. RR: respiratory rate; CI: confidence interval; SD: standard deviation

The RR was analyzed in four studies involving 136 patients. The pooled mean was 18.68 breaths per minute (95% CI: 13.68-25.52) (Figure 10), again indicating high heterogeneity (I² = 99.8%). These values suggest the preservation of spontaneous ventilation during sedation with the KD, with no consistent reports of respiratory depression.

**Figure 11:**
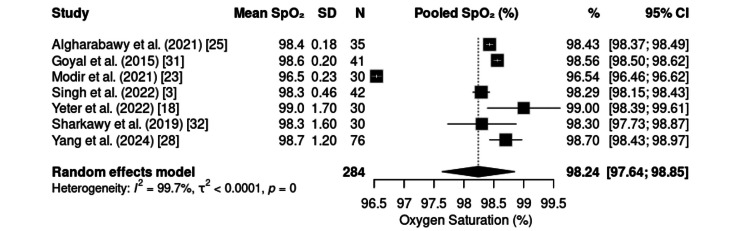
Forest plot of intraoperative SpO₂. SpO₂: oxygen saturation; CI: confidence interval; SD: standard deviation

Peripheral SpO₂ was reported in eight studies, including 315 patients, with a pooled mean of 98.24% (95% CI: 97.64-98.85) (Figure 11). All studies demonstrated adequate maintenance of oxygenation.

Cardiovascular Events

**Figure 12:**
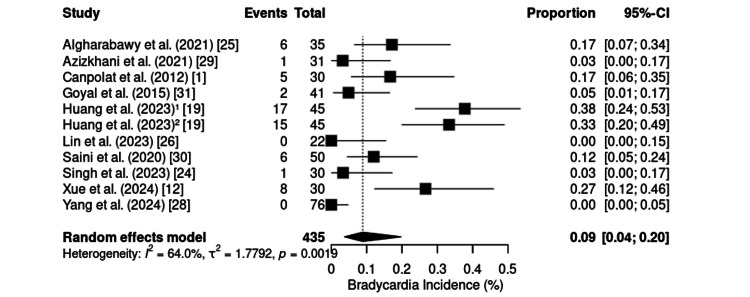
Forest plot of bradycardia incidence. CI: confidence interval

Bradycardia was reported in 11 randomized controlled trials, including 435 patients, with a pooled incidence of 9% (95% CI: 4%-20%) (Figure 12). The analysis revealed moderate to high heterogeneity (I² = 64.0%; τ² = 1.7792; p = 0.0019). The incidence ranges from 0% [26,28] to 37.8% [19], with the highest rates observed in breast cancer surgeries conducted under the KD protocol in China. Notably, gastrointestinal and emergency procedures presented lower rates, generally between 3% and 17.1%.

**Figure 13:**
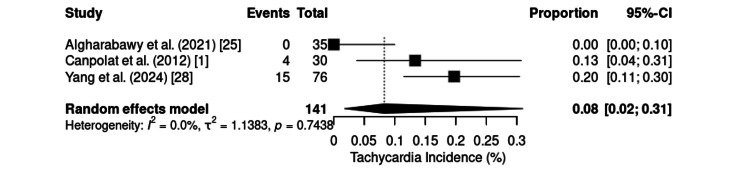
Forest plot of tachycardia incidence. CI: confidence interval

Tachycardia was observed in three RCTs involving 141 patients, with a pooled incidence of 8% (95% CI: 2%31%) and no heterogeneity (I² = 0%; τ² = 1.1383; p = 0.7438) (Figure 13). The event was absent in gastrointestinal endoscopy [25], moderate in cesarean sections (19.7%) [28], and intermediate in burn dressing procedures (13.3%) [1].

**Figure 14:**
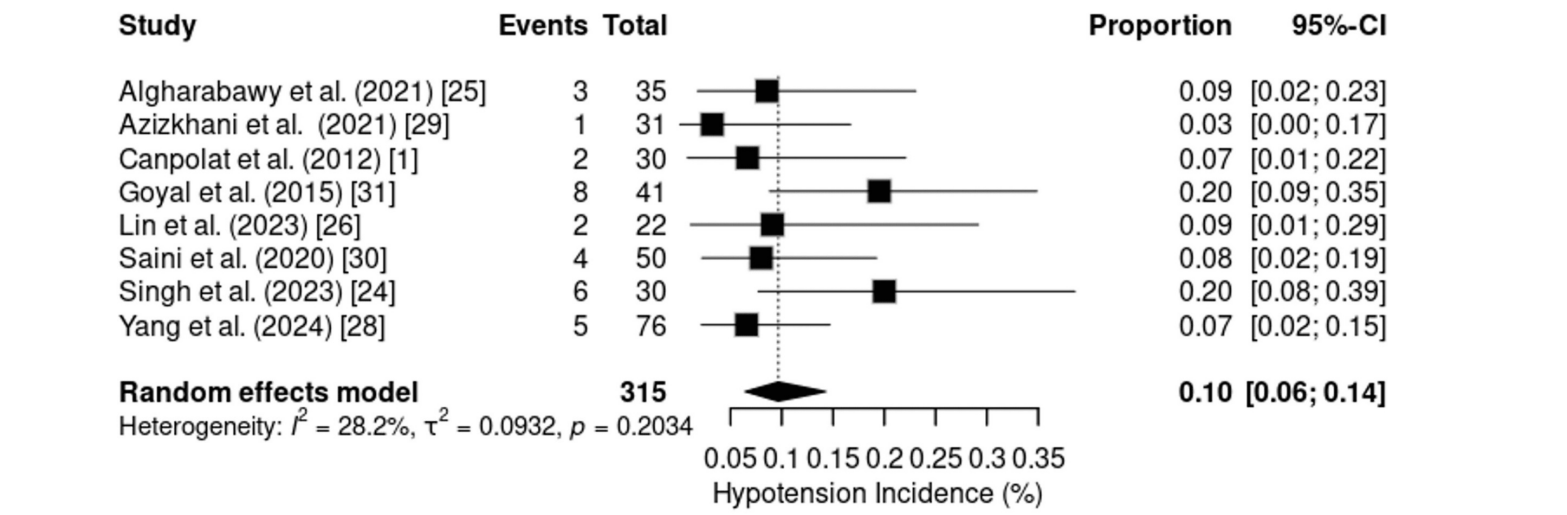
Forest plot of hypotension incidence. CI: confidence interval

Hypotension was reported in eight studies, comprising 315 patients, with a pooled incidence of 10% (95% CI:6%-14%). The analysis revealed low to moderate heterogeneity (I² = 28.2%; τ² = 0.0932; p = 0.2034) (Figure 14). The incidence ranged from 3% to 20%, with most studies reporting rates between 6.7% and 9%. Two studies [24,28] reported slightly higher rates of 20% and 7%, respectively.

**Figure 15:**
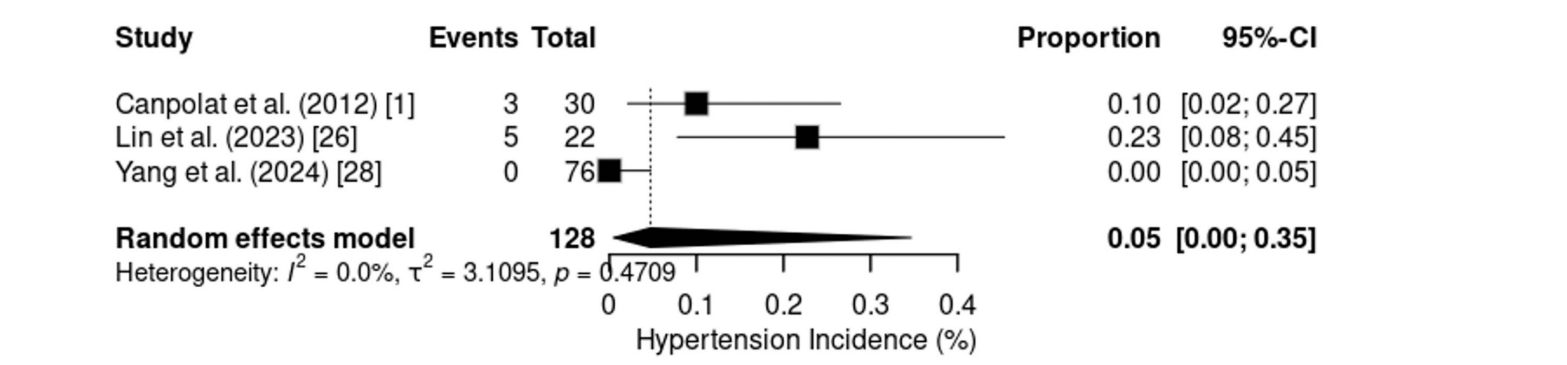
Forest plot of hypertension incidence. CI: confidence interval

Hypertension was identified in three studies, including 136 patients, with a pooled incidence of 5% (95% CI: 0%-35%) (Figure 15). No heterogeneity was detected (I² = 0%; τ² = 3.1095; p = 0.4709). The reported incidence ranges from 0% [28] to 22.7% [26], with gastrointestinal endoscopy studies reporting an intermediate value of 10% [1].

Respiratory Safety

**Figure 16:**
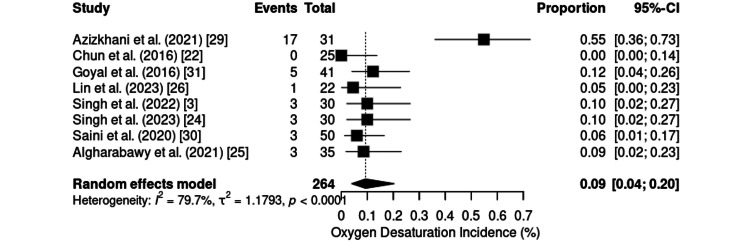
Forest plot of desaturation incidence. CI: confidence interval

Oxygen desaturation was assessed in eight RCTs, involving a total of 264 adult patients sedated with the KD combination. The pooled incidence was 9% (95% CI: 4%-20%), with significant heterogeneity (I² = 79.7%; τ² = 1.1793; p < 0.0001) (Figure 16). Individual rates varied widely, ranging from 0% during chemoport insertion [22] to 55% in painful emergency procedures in the emergency department [29], the latter reflecting the higher incidence observed in settings with greater clinical instability. These findings suggest that desaturation may occur more frequently in urgent situations characterized by heightened physiological stress.

In contrast, during elective procedures, the incidence rates were substantially lower, ranging from 4.5% to 11.9%. Notable examples include laparoscopic cholecystectomy (6%) [30], radiofrequency ablation of pulmonary tumors (4.5%) [26], and upper gastrointestinal endoscopy (8.57%) [1]. More complex endoscopic procedures, such as endoscopic ERCP, have rates between 10% and 11.9% [3,24,31]. Overall, oxygen desaturation associated with KD use is uncommon in elective and minimally invasive settings and remains within clinically acceptable limits.

Gastrointestinal Symptoms

Unlike previous reviews that opted to group postoperative nausea and vomiting (PONV) as a single adverse event to simplify analysis, the present review treated nausea and vomiting as independent outcomes. This approach was deliberately adopted for methodological reasons, as most of the included studies reported these outcomes separately, allowing for more specific and clinically meaningful estimates.

**Figure 17:**
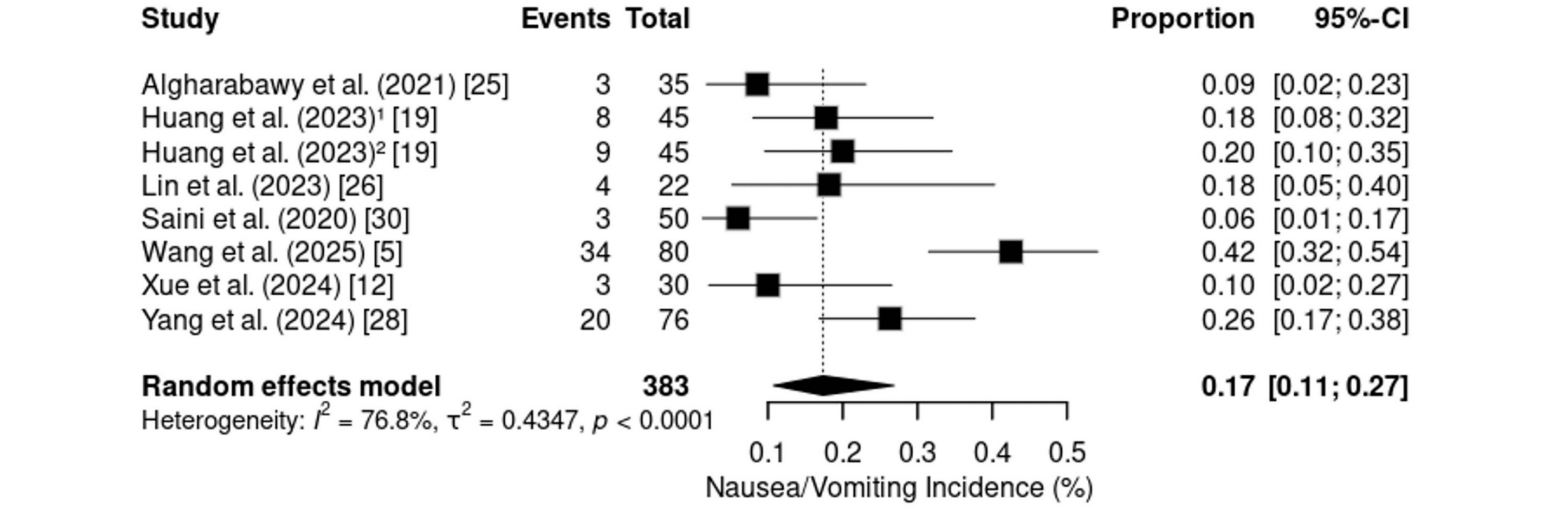
Forest plot of nausea/vomiting incidence. CI: confidence interval

The combined occurrence of nausea and vomiting was reported as a single outcome in eight RCTs involving 383 patients and had an incidence of 17% (95% CI: 11%-27%), with moderate to high heterogeneity (I² = 76.8%; τ² = 0.4347; p < 0.0001) (Figure 17). The rates ranged from 6.0% in laparoscopic cholecystectomy [30] to 42.5% in major laparoscopic abdominal surgeries [5]. Higher rates were more frequently associated with invasive abdominal or obstetric procedures, whereas lower values were observed in upper gastrointestinal endoscopy [25] and shoulder arthroscopy [12].

**Figure 18:**
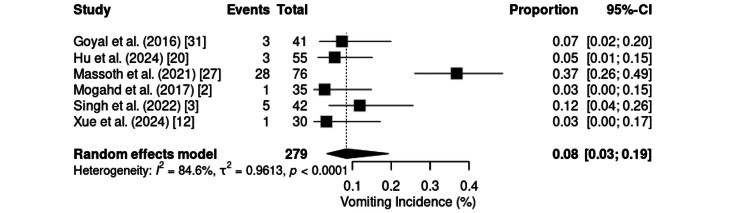
Forest plot of vomiting incidence. CI: confidence interval

Vomiting, assessed in six studies involving 279 patients, had a pooled incidence of 8% (95% CI: 3%-19%), with high heterogeneity (I² = 84.6%; τ² = 0.9613; p < 0.0001) (Figure 18). The highest incidence was reported for gynecologic laparoscopy [8] (36.8%), while most of the remaining studies reported rates less than 12%. Particularly low values were observed in cardiac procedures (2.9%) [2] and orthopedic procedures (3.3%) [12].

**Figure 19:**
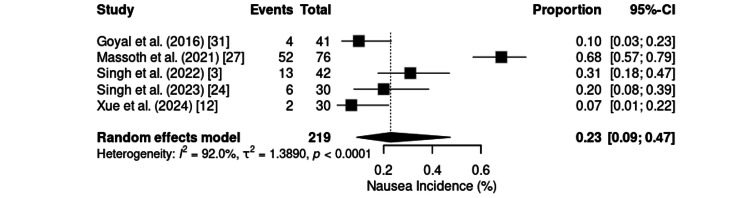
Forest plot of nausea incidence. CI: confidence interval

Nausea, analyzed independently in five randomized trials with 219 patients, had a pooled incidence of 23% (95% CI: 9%-47%) and very high heterogeneity (I² = 92.0%; τ² = 1.3890; p < 0.0001) (Figure 19). The rates ranged from 6.7% for shoulder arthroscopy [12] to 68.4% for gynecologic laparoscopy [27]. Intermediate rates were observed in ERCP procedures, with studies [3,24,31] reporting incidences between 10% and 31%.

Neuropsychiatric Effects

**Figure 20:**
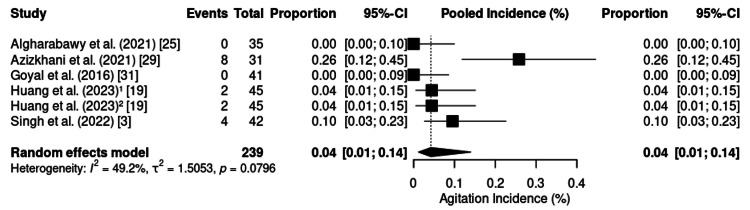
Forest plot of agitation incidence. CI: confidence interval

Agitation was reported in six randomized controlled trials (n = 239). No significant heterogeneity was observed (p = 0.39; I² = 4%; τ² = 0.01) (Figure 20), allowing for the use of a fixed-effects model. The estimated pooled incidence was 6% (95% CI: 3%-10%). Individual rates ranged from 0% [19,25,31] to 26% [29], with higher frequencies observed during painful procedures performed in emergency settings.

**Figure 21:**
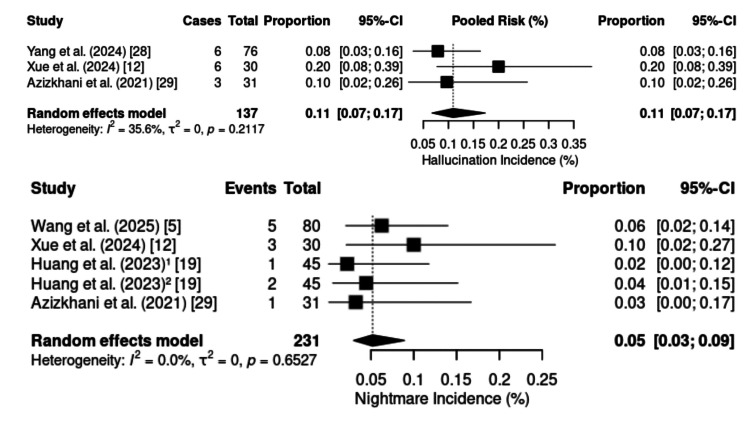
Forest plot of nightmares and hallucinations incidence. CI: confidence interval

Hallucinations were described in five RCTs (n = 313). No heterogeneity was detected (p = 0.46; I² = 0%; τ² ≈ 0), justifying the use of a fixed-effects model. The pooled incidence was 7% (95% CI: 4%-12%) (Figure 21). The highest frequency was observed during shoulder arthroscopy (20%) [12], whereas lower rates occurred in cesarean sections (8.5%) [28] and emergency procedures (10%). [29]. Nightmares were evaluated in five RCTs (n = 231), with no evidence of heterogeneity (p = 0.80; I² = 0%; τ² = 0.02). The pooled incidence was 5% (95% CI: 3%-9%) (Figure 21), ranging from 2.2% following radical mastectomy [19] to 10% during shoulder arthroscopy [12].

**Figure 22:**
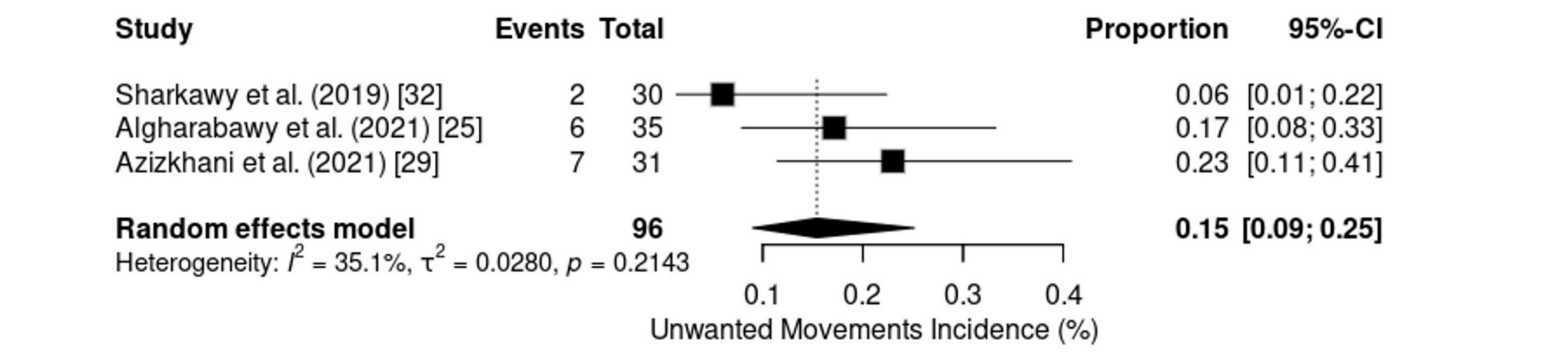
Forest plot of unwanted movements incidence. CI: confidence interval

Unwanted movements were reported in three studies (n = 96). The analysis revealed low to moderate heterogeneity (p = 0.29; I² = 20%; τ² = 0.02), and a random effects model was applied. The pooled incidence was 15% (95% CI: 9%-25%) (Figure 22), ranging from 6% for awake fiberoptic intubation [32] to 23% for emergency procedures [29].

Risk of Bias Assessment and Limitations

Randomization (D1): Sixteen of the 19 studies were rated as low risk, reflecting adequate random sequence generation and allocation concealment. In three trials [2,25,32], insufficient description of the methods introduced uncertainty regarding protection against selection bias, thereby limiting full confidence in the initial comparability of groups.

Deviations from intended interventions (D2): Most studies maintained rigor in the execution of interventions, suggesting good adherence to protocols and minimal risk of contamination across groups. Only one trial [32] provided unclear reporting regarding protocol adherence, although no evidence of systematic deviations that could compromise outcomes was identified.

Missing outcome data (D3): All studies were assessed as low risk. Losses to follow-up were rare and, when present, generally justified or balanced between groups, minimizing the likelihood of attrition bias. The overall completeness of the data strengthens the robustness of this review’s conclusions.

Measurement of outcomes (D4): In 13 studies, outcomes were consistently evaluated, often using objective parameters such as heart rate, blood pressure, sedation scores, or incidence of adverse events. However, six trials [2,3,12,18,30,32] did not clarify whether outcome assessors were blinded to group allocation or whether measurement methods were fully standardized. This lack of information increases the risk of detection bias, particularly for more subjective outcomes such as patient satisfaction and postoperative recovery.

Selection of the reported results (D5): Most studies were also judged as low risk. Nonetheless, four trials [1,2,12,32] provided insufficient details regarding blinding or standardization in outcome assessment, creating uncertainty, especially for clinically subjective variables such as sedation scores.

**Figure 23:**
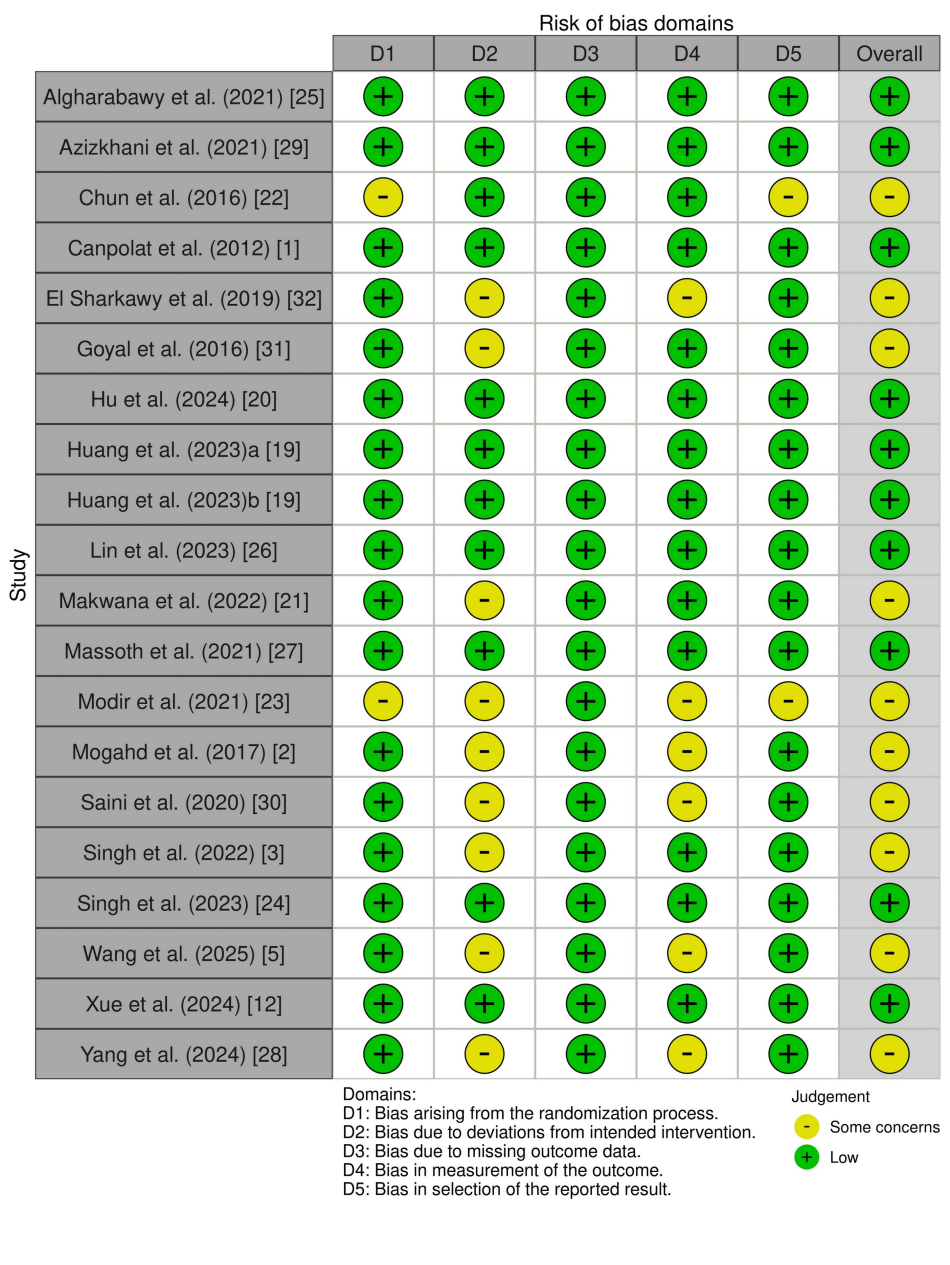
Risk of bias assessed with RoB 2. Risk of bias was evaluated across five domains. An overall risk of bias judgment is also presented for each study. Green circle with “+”: low concerns; yellow circle with “–”: some concerns. RoB 2: Cochrane Risk-of-Bias 2 tool.

The trials analyzed demonstrated methodological robustness, with a low risk of bias in most domains (Figure 23). Nevertheless, weaknesses in randomization, blinding, and standardization of outcome assessment warrant caution, particularly in the interpretation of subjective endpoints.

Limitations

Beyond the risk of bias, this review is characterized by methodological heterogeneity among studies, reflected in variations in anesthetic regimens, types of procedures, and patient populations. This variability likely contributed to the dispersion of the results and limited the generalizability of the pooled estimates. Differences in the dosing and administration protocols of ketamine-dexmedetomidine may also have influenced the magnitude of the observed effects.

Upcoming trials should prioritize standardized protocols, rigorous blinding procedures, and extended follow-up to increase the reliability of clinical recommendations. Meta-analyses based on individual patient data represent a promising strategy to reduce uncertainty and increase the applicability of findings across diverse surgical settings

Discussion

Clinical Effectiveness

Postoperative pain and analgesic effectiveness: Effective postoperative pain management is a cornerstone of recovery and patient satisfaction. The KD combination consistently reduces pain scores in the early postoperative period, particularly in minimally invasive procedures such as shoulder arthroscopy and laparoscopic cholecystectomy. This benefit reflects the multimodal analgesia of both agents, since dexmedetomidine attenuates nociceptive transmission via central and spinal α₂-adrenergic receptors, while ketamine prevents central sensitization and opioid-induced hyperalgesia. Importantly, the observed synergism arises from the complementary modulation of excitatory and inhibitory pain pathways, which explains the enhanced analgesia achieved without significant respiratory compromise [14,16,33].

It is important to note that, due to the heterogeneity of comparators among the included trials, this meta-analysis adopted a single-arm synthesis focused exclusively on KD outcomes. This approach allows for an internally consistent evaluation of efficacy and safety while minimizing interpretive bias introduced by non-equivalent control interventions or variable adjunctive drugs.

The VAS scores were predominantly low in the first hours after surgery, indicating greater comfort and a reduced need for rescue analgesia. These clinical effects are consistent with the pharmacodynamic complementarity of KD and align with current multimodal analgesia guidelines, which emphasize minimizing opioid exposure without compromising pain relief. In addition, the hemodynamic balance achieved by the opposing sympathetic effects of ketamine and dexmedetomidine may further contribute to patient stability during emergence and recovery [27,34].

Among more extensive procedures, such as radical mastectomy, KD alone is less effective, with higher pain scores and a greater incidence of residual pain [19,33,35]. Here, the central mechanisms of both drugs remain active, but the greater nociceptive burden requires integration into broader multimodal regimens, including nonsteroidal anti-inflammatory drugs and regional nerve blocks. Notably, our analysis revealed no consistent associations between KD dose, surgery duration, and increased pain, suggesting a stable analgesic effect across different contexts. Such consistency supports the idea that ketodex’s efficacy is primarily related to its central neuromodulatory effects rather than procedure-dependent pharmacokinetics [8,34].

Evidence also indicates that higher doses of ketamine (≥0.5 mg/kg) and dexmedetomidine (≥0.5 μg/kg) prolong analgesia and maintain lower VAS scores, although at the cost of greater risks such as bradycardia and prolonged sedation. Thus, protocol selection should balance efficacy with safety, adapting dosing to procedure type and patient profile. This balance underscores the clinical relevance of using subanesthetic ketamine doses and moderate α₂-agonist titration to optimize analgesia while minimizing adverse effects [8,33].

In summary, the KD combination offers reliable immediate postoperative analgesia, with the greatest benefit in minimally invasive surgeries. Their role in major procedures is best realized as part of multimodal strategies, enabling personalized pain control while supporting the broader goal of reducing opioid dependence in modern anesthesia practice [8,11,36].

Physician and Patient Satisfaction

Satisfaction assessment is a key outcome in sedation protocols, as it reflects both the patient’s subjective experience and the professional perception of the medical team. In this analysis, the predominance of “excellent” patient ratings suggests that the KD provides a well-tolerated and positively perceived sedative experience. The pharmacological characteristics of dexmedetomidine, which induces more natural sleep-like sedation, and ketamine, which ensures robust analgesia and prevents negative recall, contribute to minimizing discomfort and incidental pain, thereby increasing overall patient acceptance [14,16,33,37].

In contrast, physician satisfaction was more conservative, with “adequate” ratings occurring more frequently than “excellent” ratings. This divergence reflects distinct evaluative criteria: while patients prioritize comfort, the absence of pain, and perceived stability, physicians tend to emphasize induction predictability, recovery speed, and logistical efficiency. Although the KD ensures safety and stability, these factors may temper physicians’ perceptions of “excellence” in high-demand environments, where workflow efficiency is highly valued [36,37].

Overall, this discrepancy should not be interpreted as a limitation of the KD but as a consequence of different expectations between patients and physicians. Whereas patients focus on subjective well-being, physicians weigh operational and intraoperative parameters [36,37]. Given the growing emphasis on patient-centered metrics in anesthetic practice, the consistently high patient satisfaction with the KD is particularly relevant, supporting its role as a balanced strategy between safety and the user experience.

Recovery and Post-anesthesia Care Stay Times

In the analysis of the included studies, the induction time with the KD combination was consistently rapid, ranging from 6.3 minutes [27] to 8.0 minutes [25]. From a pharmacological standpoint, the rapid induction primarily reflects ketamine’s high lipophilicity and rapid central nervous system penetration, which results in fast dissociative anesthesia. Dexmedetomidine’s slower onset does not significantly delay induction, as the initial ketamine effect predominates. Additionally, the combination allows the use of subanesthetic ketamine doses, which mitigates cardiovascular stimulation while maintaining swift onset. This profile makes the KD suitable for both ambulatory and inpatient procedures and clinically comparable to propofol or midazolam while providing greater hemodynamic stability [3,25].

Recovery times with the KD showed greater variability, ranging from 6.5 minutes [26] to 36.6 minutes [1]. This dispersion is closely linked to procedure characteristics and anesthetic protocols rather than a limitation of the KD itself. This variability can be explained by the interplay between ketamine and dexmedetomidine. Ketamine’s dissociative effect is short-acting and rapidly metabolized, facilitating early return of consciousness. Dexmedetomidine provides longer-lasting, physiologic-like sedation via central α₂-adrenoceptor agonism, which may modestly prolong recovery but contributes to smooth awakening and autonomic stability. Although meta-analyses suggest that other combinations, such as ketofol (ketamine + propofol), may allow for slightly faster recovery, the differences are small and not clinically relevant in terms of safety, respiratory events, or cardiovascular stability [22,38].

The PACU stay demonstrated the widest variability among the time-based outcomes and was strongly influenced by procedural type, postoperative analgesia, and population characteristics. The durations ranged from 26 minutes [19] to 69 minutes [27]. This suggests that PACU length is determined more by contextual and surgical factors than by the sedative regimen itself [1,27].

From a pharmacological perspective, this temporal profile reflects the complementary properties of the KD. Ketamine, with its rapid-onset and short-duration dissociative action, contributes to swift induction and early return of consciousness, whereas dexmedetomidine, with longer-acting sedative effects that mimic physiological sleep, may modestly prolong recovery but enhances autonomic stability, keeping awakening times compatible with safe clinical practice [15,33].

Although the absence of direct comparators limits cross-protocol inferences, the internal consistency of the time-related outcomes across trials supports the reproducibility of KD pharmacodynamics in adult anesthesia. This reinforces the methodological choice of analyzing KD as an independent intervention, rather than against heterogeneous regimens that would confound temporal metrics.

In summary, KD ensures rapid induction, predictable recovery, and context-adjusted PACU stay durations without clinically significant delays, supporting its use across different procedural settings. Individualized titration and dose adjustment remain essential to optimize the balance between safety and efficiency.

Safety Profile and Adverse Events

Hemodynamic stability:* *The hemodynamic profile of KD reflects the interaction of opposing yet complementary mechanisms. Dexmedetomidine reduces sympathetic tone and enhances vagal activity, predisposing patients to bradycardia and hypotension, whereas ketamine exerts an indirect sympathomimetic effect by inhibiting catecholamine reuptake, leading to increased blood pressure and heart rate. When administered together, these mechanisms balance each other, mitigating extreme fluctuations and promoting a more predictable hemodynamic pattern, in which vital signs are more likely to remain within physiological limits [15,33].

This pharmacodynamic antagonism provides a form of “mutual protection,” reducing both the hypotension and bradycardia associated with α₂-agonists and the hypertension and tachycardia commonly induced by ketamine alone. Such balance is particularly advantageous in patients with autonomic instability, during prolonged procedures, or in sedation settings outside the operating room, where hemodynamic predictability and preservation of spontaneous ventilation are critical [11,36].

In the present meta-analysis, the mean values for blood pressure, heart rate, respiratory rate, and peripheral oxygen saturation generally remained within clinically stable ranges, despite considerable heterogeneity across studies. However, this stability is not absolute: clinically significant bradycardia has been reported after rapid bolus or high doses of dexmedetomidine that may cause abrupt central α₂ activation, whereas hypertensive peaks and tachycardia may occur with higher ketamine doses amplifying catecholamine release and sympathetic tone or intense nociceptive stimulation, such as burn care or cesarean sections. Thus, dosage, administration speed, procedural characteristics, and individual autonomic profiles are key modulators of the final response and help explain the variability reported across trials [11,33].

In summary, the findings of this meta-analysis support the current evidence that the KD promotes intraoperative hemodynamic stability and respiratory preservation, confirming its role as a safe and effective sedation regimen, which is particularly beneficial during procedures where rapid autonomic shifts are expected [3,11,36,38,39].

Cardiovascular Events

Despite the apparent hemodynamic stability of the KD, bradycardia has emerged as the most frequently reported cardiovascular event. Typically, mild and transient, its occurrence is influenced by the type of procedure, patient autonomic profile, and the use of other agents. However, it may be more pronounced in settings of heightened vagal tone, such as during deep sedation or regional anesthesia, particularly in mastectomies. This variability underscores that bradycardia is highly context-dependent. Whether prophylactic atropine should be considered remains controversial, but the need for vigilant hemodynamic monitoring is consistently emphasized [10,16,20,27,28,34,40].

Hypotension, although less prevalent, has also been reported and is usually linked to the central vasodilatory effect of dexmedetomidine at infusion onset. It is generally mild, self-limiting, and rarely requires pharmacological intervention. Its incidence tends to be lower in procedures associated with sympathetic activation, reinforcing the influence of the clinical context [19,27,33,37].

Tachycardia and hypertension, which are commonly associated with ketamine alone, were less common with the KD combination. Tachycardia is mainly observed in procedures involving intense nociceptive stimuli, such as cesarean section and burn dressing changes, reflecting residual sympathomimetic effects [24,35,37].

Hypertension is rare and is usually related to infusion dynamics or interindividual variability [41].

Overall, the KD was associated with a safe and predictable cardiovascular profile, with a low incidence of severe hemodynamic complications. Bradycardia is the most common event but is typically mild and manageable. Hypotension, tachycardia, and hypertension are less common, often context-dependent, and rarely severe. These findings highlight the importance of vigilant hemodynamic monitoring, particularly in patients with increased cardiovascular risk or who are undergoing high-intensity procedures [11,36].

Respiratory Safety

The KD is a sedative approach that provides deep anesthesia while preserving respiratory function. It typically maintains ventilatory drive and airway tone, with oxygen desaturation occurring only rarely in elective settings. This synergy ensures a stable ventilatory profile, making KD particularly valuable in procedures that require deep sedation without intubation [33,38].

In the present analysis, oxygen desaturation was an infrequent event during elective procedures performed under controlled clinical conditions. Interventions such as upper gastrointestinal endoscopies, laparoscopic cholecystectomies, and pulmonary radiofrequency ablations consistently resulted in a low incidence of respiratory events, supporting the respiratory safety of the KD protocol in these scenarios. These findings are in agreement with current evidence, which demonstrates a lower incidence of hypoxemia and apnea with the KD than with other sedative combinations, such as propofol-fentanyl or ketofol, particularly outpatient sedation and diagnostic procedures. In addition, the opioid-sparing effect of dexmedetomidine likely contributes to the preservation of ventilatory stability [3,14,15,19,27].

However, in emergency contexts, the pattern differs substantially, as desaturation during painful and urgent procedures indicates that the respiratory risk associated with KD is not uniform but is strongly influenced by the clinical setting. Factors such as limited patient preparation, higher baseline physiological stress, and the need for rapid interventions appear to increase the likelihood of adverse respiratory events, in contrast with the consistently low and clinically acceptable rates observed in elective and well-controlled procedures. This highlights that the safety profile of KD should be interpreted as context dependent, with respiratory complications being driven less by the intrinsic pharmacology of the agents and more by situational determinants such as patient instability, procedure urgency, and inadequate pre-sedation optimization [1,17,28,29,39].

Notably, only a limited number of studies have specifically evaluated KD sedation in emergency scenarios; thus, while current evidence suggests a greater vulnerability to hypoxemia in these settings, further confirmation from larger and context-focused investigations is needed. In this context, recent reviews agree that adverse respiratory events during out-of-operating-room sedation are more closely related to contextual variables than to the sedative regimen itself. Accordingly, the KD can be considered a safe option for elective procedures, whereas its application in emergencies warrants heightened vigilance, closer monitoring, and tailored precautionary strategies until more robust evidence becomes available [9,26,39,42].

These findings illustrate the value of a single-arm synthesis for safety assessment, since heterogeneous comparator arms (often including opioids or benzodiazepines) could obscure the intrinsic respiratory profile of KD. Focusing on KD-only data provides a clearer understanding of its ventilatory stability across elective and emergency contexts.

Overall, the evidence reinforces that the KD results in a favorable respiratory safety profile, particularly in elective settings under appropriate monitoring. Nevertheless, its use in emergencies or in patients with reduced ventilatory reserve requires continuous vigilance, systematic oxygenation monitoring, and preparedness for prompt ventilatory support when necessary [11,36].

Gastrointestinal Symptoms

The gastrointestinal effects of the KD are intermediate: ketamine can stimulate the chemoreceptor trigger zone and central vomiting pathways via NMDA receptor antagonism, which may tends to increase nausea and vomiting, whereas dexmedetomidine counteracts these effects by reducing sympathetic activity and attenuating autonomic excitability. Overall, the KD shows better tolerability than opioid-based regimens do, although it does not match the antiemetic profile of propofol [3,13,18,33,38,41].

In clinical practice, this balance translates into favorable gastrointestinal tolerability in most scenarios, particularly during less invasive procedures, where the incidence of nausea and vomiting tends to be mild and self-limiting. Conversely, in contexts of greater visceral stimulation, such as gynecologic or abdominal laparoscopies, vulnerability to the emetic response remains evident, indicating that the risk is not solely pharmacological but also conditioned by the nature of the procedure and the patient’s baseline profile [8,16,34,37,40].

Another relevant clinical consideration is the distinction between nausea and vomiting. Under KD, nausea is more prevalent and multifactorial, often occurring even in minor interventions, whereas vomiting is typically restricted to settings of greater procedural aggressiveness or pronounced autonomic stress [28,36]. Importantly, these events have different clinical weights: nausea, although less severe, substantially compromises patient comfort and subjective recovery, whereas vomiting has more significant implications, including bronchoaspiration risk, marked distress, and potential delay in hospital discharge. Recognizing this distinction is crucial, as it enables targeted preventive strategies that address both patient well-being and safety outcomes [2,11].

These findings are consistent with recent literature highlighting dexmedetomidine as a significant reducer of PONV, largely because of its opioid-sparing properties [4]. Although the KD does not achieve the antiemetic potency of propofol, it provides stable sedation, effective analgesia, and respiratory safety, making it a viable option, particularly in elective procedures requiring hemodynamic stability [11].

Overall, the evidence suggests that the KD presents a balanced gastrointestinal profile, offering advantages over highly emetogenic regimens while maintaining stable sedation and adequate analgesia. Nonetheless, the potential for residual manifestations, especially in surgeries involving high visceral stimulation, warrants heightened vigilance, including prophylactic antiemetics in at-risk patients, strict monitoring, and individualized management according to the type of intervention [19,21,27,39]. As in the cardiovascular domain, the key to maximizing the benefits of this protocol lies in acknowledging its limitations and tailoring its application to the clinical realities of each scenario.

Neuropsychiatric Effects

The neuropsychiatric effects associated with ketamine, such as agitation, hallucinations, nightmares, and unwanted movements, represent one of the main limitations of its use as a sole agent in sedation and anesthesia. These manifestations stem from its dissociative action and antagonism of NMDA receptors, which may disrupt excitatory glutamatergic signaling in cortical and limbic circuits elicit intensified perceptual experiences and distressing emergence phenomena for patients. These effects are dose-dependent and more pronounced when ketamine is used as a sole sedative agent [1,12,33].

In contrast, when combined with dexmedetomidine, the KD provides a smoother transition between sedation and wakefulness, mitigating the psychomimetic effects of ketamine without prolonging discharge from the recovery unit, through reduction of the noradrenergic outflow from the locus coeruleus, which attenuates ketamine-induced cortical hyperexcitability and psychomimetic phenomena. This pharmacologic synergy allows a calmer emergence and more predictable recovery, supporting the clinical observations of improved patient comfort. Patients frequently report greater comfort and reduced disorientation upon awakening than those receiving ketamine-only regimens do, reinforcing its value in ambulatory settings and short-duration procedures [30,33,37,38].

In our analysis, the incidence of hallucinations, nightmares, and headaches associated with KD was relatively low, corroborating the literature that points to a favorable neuropsychiatric profile for this combination. This finding supports the hypothesis that dexmedetomidine not only attenuates the dissociative effects of ketamine but also provides a more predictable and acceptable sedation experience for both patients and healthcare teams [2,38].

Nevertheless, we observed that the occurrence of unwanted movements was relatively greater, particularly during painful procedures or in emergency settings. Although the incidence may appear relatively low, it remains clinically relevant and deserves attention, since such reactions can interfere with the procedure and increase the risk of complications, directly impacting both patient safety and procedural efficacy. This phenomenon may reflect a limitation of the combined regimen in fully suppressing motor responses to intense nociceptive stimuli, especially when suboptimal dosing strategies are employed [8,12,17,36]. Therefore, careful adjustment of doses is essential, and in specific contexts, particularly when immobility is critical, the association with neuromuscular blockade may be considered an additional strategy to minimize adverse effects.

In summary, the KD has emerged as a promising strategy to overcome the neuropsychiatric limitations of ketamine alone, balancing analgesia and hemodynamic stability. Another relevant point is that the KD consistently promotes a smoother transition between sedation and wakefulness. Compared with patients receiving ketamine-based regimens, patients often report greater comfort and less disorientation during recovery, further strengthening its role in outpatient and short-duration procedures [11,30,38,37].

## Conclusions

Our meta-analysis revealed that the KD is generally effective and safe for sedation in surgical and nonsurgical procedures in elective settings, with consistent benefits across most studies despite some methodological heterogeneity and isolated concerns regarding randomization, blinding, and outcome measurement. Nonetheless, evidence suggests that adverse events occur more frequently in emergency contexts, indicating that the safety profile of the KD is not uniform across all clinical scenarios and warrants particular caution in unstable or urgent settings. Future research should prioritize large, randomized, multicenter trials with standardized dosing regimens, rigorous blinding, and long-term follow-up to confirm these findings and optimize their use across diverse clinical settings.

## References

[1] Canpolat DG, Esmaoglu A, Tosun Z, Akn A, Boyaci A, Coruh A (2012). Ketamine-propofol vs ketamine-dexmedetomidine combinations in pediatric patients undergoing burn dressing changes. J Burn Care Res.

[2] Mogahd MM, Mahran MS, Elbaradi GF (2017). Safety and efficacy of ketamine-dexmedetomidine versus ketamine-propofol combinations for sedation in patients after coronary artery bypass graft surgery. Ann Card Anaesth.

[3] Singh A, Iyer KV, Maitra S (2022). Ketamine and dexmedetomidine (Keto-dex) or ketamine and propofol (Keto-fol) for procedural sedation during endoscopic retrograde cholangiopancreatography: which is safer? A randomized clinical trial. Indian J Gastroenterol.

[4] Tobias JD (2012). Dexmedetomidine and ketamine: an effective alternative for procedural sedation?. Pediatr Crit Care Med.

[5] Wang W, Chen Y, Li G, Chen Y, Wu J, Shi Y, Zhong M (2025). The opioid-sparing effects of intraoperative esketamine combined with dexmedetomidine during laparoscopic major abdominal surgery: a randomized controlled double-blind trial. Drug Des Devel Ther.

[6] Jin S, Liang DD, Chen C, Zhang M, Wang J (2017). Dexmedetomidine prevent postoperative nausea and vomiting on patients during general anesthesia: a PRISMA-compliant meta analysis of randomized controlled trials. Medicine (Baltimore).

[7] Kim HJ, Kim YJ, Lee J (2025). Comparison of the recovery profiles of propofol, dexmedetomidine, and remimazolam for intraoperative sedation in patients undergoing upper limb surgery under brachial plexus blockade: a randomized controlled trial. Can J Anaesth.

[8] Kurdi MS, Theerth KA, Deva RS (2014). Ketamine: current applications in anesthesia, pain, and critical care. Anesth Essays Res.

[9] Pichot C, Longrois D, Ghignone M, Quintin L (2012). [Dexmedetomidine and clonidine: a review of their pharmacodynamy to define their role for sedation in intensive care patients]. Ann Fr Anesth Reanim.

[10] Bajwa SJ (2021). Dexmedetomidine and ketamine - comrades on an eternal journey!. Indian J Anaesth.

[11] Kaye AD, Chernobylsky DJ, Thakur P (2020). Dexmedetomidine in enhanced recovery after surgery (ERAS) protocols for postoperative pain. Curr Pain Headache Rep.

[12] Xue Z, Yan C, Liu Y (2024). Opioid-free anesthesia with esketamine-dexmedetomidine versus opioid-based anesthesia with propofol-remifentanil in shoulder arthroscopy: a randomized controlled trial. BMC Surg.

[13] Geng ZY, Liu YF, Wang SS, Wang DX (2016). Intra-operative dexmedetomidine reduces early postoperative nausea but not vomiting in adult patients after gynaecological laparoscopic surgery: a randomised controlled trial. Eur J Anaesthesiol.

[14] Mion G, Villevieille T (2013). Ketamine pharmacology: an update (pharmacodynamics and molecular aspects, recent findings). CNS Neurosci Ther.

[15] Vekhova KA, Namiot ED, Jonsson J, Schiöth HB (2025). Ketamine and esketamine in clinical trials: FDA-approved and emerging indications, trial trends with putative mechanistic explanations. Clin Pharmacol Ther.

[16] Zanos P, Moaddel R, Morris PJ (2018). Ketamine and ketamine metabolite pharmacology: insights into therapeutic mechanisms. Pharmacol Rev.

[17] Lin J, Figuerado Y, Montgomery A (2021). Efficacy of ketamine for initial control of acute agitation in the emergency department: a randomized study. Am J Emerg Med.

[18] Yeter T, Onur Gönen A, Türeci E (2022). Dexmedetomidine vs propofol as an adjunct to ketamine for electroconvulsive therapy anaesthesia. Turk J Anaesthesiol Reanim.

[19] Huang Z, Liu N, Hu S, Ju X, Xu S, Wang S (2023). Effect of dexmedetomidine and two different doses of esketamine combined infusion on the quality of recovery in patients undergoing modified radical mastectomy for breast cancer - a randomised controlled study. Drug Des Devel Ther.

[20] Hu F, Wang Q, Yang Y, Liu Y (2024). The impact of esketamine combined with dexmedetomidine on laparoscopic gallbladder surgery: a randomized controlled trial. Altern Ther Health Med.

[21] Makwana M, Pathak B, Panchal NN (2022). Preemptive analgesysation with ketamine-dexmedetomidine versus ketamine-propofol in upper limb surgeries under supraclavicular brachial plexus block: a randomized controlled trial. Indian J Anaesth.

[22] Chun EH, Han MJ, Baik HJ (2016). Dexmedetomidine-ketamine versus dexmedetomidine-midazolam-fentanyl for monitored anesthesia care during chemoport insertion: a prospective randomized trial. BMC Anesthesiol.

[23] Modir H, Moshiri E, Yazdi B, Kamalpour T, Goodarzi D, Mohammadbeigi A (2020). Efficacy of dexmedetomidine-ketamine vs. fentanylketamine on saturated oxygen, hemodynamic responses and sedation in cystoscopy: a doubleblinded randomized controlled clinical trial. Med Gas Res.

[24] Singh J, Pathania J, Bodh V, Sharma R, Kumar R, Sharma B (2023). Etomidate-ketamine versus dexmedetomidine-ketamine for entropy-guided procedural sedation during endoscopic retrograde cholangiopancreatography procedures: a randomized single blind study. Indian J Gastroenterol.

[25] Algharabawy WS, Abusinna RG, AbdElrahman TN (2021). Dexmedetomidine-ketamine versus propofol-ketamine for sedation during upper gastrointestinal endoscopy in hepatic patients (a comparative randomized study). Egypt J Anaesth.

[26] Lin Z, Li S, Zhou Y (2023). A comparative study of esketamine-dexmedetomidine and sufentanil-dexmedetomidine for sedation and analgesia in lung tumor percutaneous radiofrequency ablation (PRFA): a randomized double-blind clinical trial. BMC Anesthesiol.

[27] Massoth C, Schwellenbach J, Saadat-Gilani K, Weiss R, Pöpping D, Küllmar M, Wenk M (2021). Impact of opioid-free anaesthesia on postoperative nausea, vomiting and pain after gynaecological laparoscopy - a randomised controlled trial. J Clin Anesth.

[28] Yang JR, Li YY, Ran TJ, Lin XY, Xu JY, Zhou SL, Huang PJ (2024). Esketamine combined with dexmedetomidine to reduce visceral pain during elective cesarean section under combined spinal-epidural anesthesia: a double-blind randomized controlled study. Drug Des Devel Ther.

[29] Azizkhani R, Kouhestani S, Heydari F, Majidinejad S (2021). A comparative study of dexmedetomidine and propofol to prevent recovery agitation in adults undergoing procedural sedation with ketamine: a randomized double-blind clinical trial. Am J Emerg Med.

[30] Saini H, Angral R, Sharma S, Sharma RR, Kumar R (2020). Comparision of dexmedetomidine and propofol in patients undergoing laparoscopic cholecystectomy under spinal anesthesia. Anesth Essays Res.

[31] Goyal R, Hasnain S, Mittal S, Shreevastava S (2016). A randomized, controlled trial to compare the efficacy and safety profile of a dexmedetomidine-ketamine combination with a propofol-fentanyl combination for ERCP. Gastrointest Endosc.

[32] El Sharkawy RA (2019). Efficacy of adding low-dose ketamine to dexmedetomidine versus low-dose ketamine and propofol for conscious sedation in patients undergoing awake fiber-optic intubation. Anesth Essays Res.

[33] Weerink MA, Struys MM, Hannivoort LN, Barends CR, Absalom AR, Colin P (2017). Clinical pharmacokinetics and pharmacodynamics of dexmedetomidine. Clin Pharmacokinet.

[34] Chou R, Gordon DB, de Leon-Casasola OA (2016). Management of postoperative pain: a clinical practice guideline from the American Pain Society, the American Society of Regional Anesthesia and Pain Medicine, and the American Society of Anesthesiologists' Committee on Regional Anesthesia, Executive Committee, and Administrative Council. J Pain.

[35] Caruso K, Tyler D, Lyden A (2021). Ketamine for pain management: a review of literature and clinical application. Orthop Nurs.

[36] Khorsand S, Karamchandani K, Joshi GP (2022). Sedation-analgesia techniques for nonoperating room anesthesia: an update. Curr Opin Anaesthesiol.

[37] Nielsen JR (2021). Sedation safety and satisfaction. Anesth Analg.

[38] Nishizawa T, Suzuki H, Hosoe N, Ogata H, Kanai T, Yahagi N (2017). Dexmedetomidine vs propofol for gastrointestinal endoscopy: a meta-analysis. United European Gastroenterol J.

[39] Fonseca FJ, Ferreira L, Rouxinol-Dias AL, Mourão J (2023). Effects of dexmedetomidine in non-operating room anesthesia in adults: a systematic review with meta-analysis. Braz J Anesthesiol.

[40] Karunarathna I (2025). The role of atropine in modern medicine: indications, administration, and clinical outcomes. Uva Clin Anaesth Intensive Care.

[41] Zhang W, Wang R, Li B, Zhao Y, Liu X, Yuan J (2022). The effect of dexmedetomidine on postoperative nausea and vomiting in patients undergoing thoracic surgery: a meta-analysis of randomized controlled trials. Front Surg.

[42] Barbic D, Andolfatto G, Grunau B (2021). Rapid agitation control with ketamine in the emergency department: a blinded, randomized controlled trial. Ann Emerg Med.

[43] Page MJ, McKenzie JE, Bossuyt PM (2021). The PRISMA 2020 statement: an updated guideline for reporting systematic reviews. BMJ.

[44] Cumpston M, Li T, Page MJ, Chandler J, Welch VA, Higgins JP, Thomas J (2019). Updated guidance for trusted systematic reviews: a new edition of the Cochrane Handbook for Systematic Reviews of Interventions. Cochrane Database Syst Rev.

[45] Sterne JA, Savović J, Page MJ (2019). RoB 2: a revised tool for assessing risk of bias in randomised trials. BMJ.

[46] Wan X, Wang W, Liu J, Tong T (2014). Estimating the sample mean and standard deviation from the sample size, median, range and/or interquartile range. BMC Med Res Methodol.

